# Acetyl-CoA carboxylase 1 inhibition increases Treg metabolism and graft-versus-host disease treatment efficacy via mitochondrial fusion

**DOI:** 10.1172/JCI182480

**Published:** 2025-09-30

**Authors:** Cameron McDonald-Hyman, Ethan G. Aguilar, Ewoud B. Compeer, Michael C. Zaiken, Stephanie Y. Rhee, Fathima A. Mohamed, Jemma H. Larson, Michael L. Loschi, Christopher Lees, Govindarajan Thangavelu, Margaret L. Sleeth, Kyle D. Smith, Jennifer S. Whangbo, Jerome Ritz, Tim D. Sparwasser, Roddy S. O’Connor, Peter A. Crawford, Jeffrey C. Rathmell, Leslie S. Kean, Robert Zeiser, Keli L. Hippen, Michael L. Dustin, Bruce R. Blazar

**Affiliations:** 1 Division of Hematology/Oncology/Transplantation, Department of Medicine, and; 2 Division of Blood and Marrow Transplantation, Department of Pediatrics, Masonic Cancer Center, University of Minnesota, Minneapolis, Minnesota, USA.; 3 Kennedy Institute of Rheumatology, Nuffield Department of Orthopaedics, Rheumatology and Musculoskeletal Sciences, University of Oxford, Oxford, United Kingdom.; 4 Division of Hematology/Oncology, Boston Children’s Hospital, Dana-Farber Cancer Institute, and; 5 Division of Hematologic Malignancies, Dana-Farber Cancer Institute, Harvard Medical School, Boston, Massachusetts, USA.; 6 Institute of Medical Microbiology and Hygiene, University Medical Center of the Johannes Gutenberg University, Mainz, Germany.; 7 Center for Cellular Immunotherapies, Department of Pathology and Laboratory Medicine, Perelman School of Medicine at the University of Pennsylvania, Philadelphia, Pennsylvania, USA.; 8 Division of Molecular Medicine, Department of Medicine, University of Minnesota, Minneapolis, Minnesota, USA.; 9 Vanderbilt Center for Immunobiology, Department of Pathology, Microbiology, and Immunology, Vanderbilt University Medical Center, Nashville, Tennessee, USA.; 10 Department of Medicine I, Medical Center–University of Freiburg, Faculty of Medicine, Albert Ludwigs University, Freiburg, Germany.

**Keywords:** Immunology, Metabolism, Bone marrow transplantation, Mitochondria, T cells

## Abstract

Tregs are critical for maintaining immune homeostasis, and their adoptive transfer can treat murine inflammatory disorders. In patients, Treg therapies have been variably efficacious. Therefore, new strategies to enhance Treg therapeutic efficacy are needed. Tregs predominantly depend on oxidative phosphorylation (OXPHOS) for energy and suppressive function. Fatty acid oxidation (FAO) contributes to Treg OXPHOS and can be important for Treg “effector” differentiation, but FAO activity is inhibited by coordinated activity of the isoenzymes acetyl-CoA carboxylase-1 and -2 (ACC1 and ACC2). Here, we show that small-molecule inhibition or Treg-specific genetic deletion of ACC1 significantly increases Treg suppressive function in vitro and in mice with established chronic graft-versus-host disease. ACC1 inhibition skewed Tregs toward an “effector” phenotype and enhanced FAO-mediated OXPHOS, mitochondrial function, and mitochondrial fusion. Inhibiting mitochondrial fusion diminished the effect of ACC1 inhibition. Reciprocally, promoting mitochondrial fusion, even in the absence of ACC1 modulation, resulted in a Treg functional and metabolic phenotype similar to that seen with ACC1 inhibition, indicating a key role for mitochondrial fusion in Treg-suppressive potency. Ex vivo–expanded, ACC1 inhibitor–treated human Tregs similarly augmented suppressor function, as observed with murine Tregs. Together, these data suggest that ACC1 manipulation may be exploited to modulate Treg function in patients.

## Abstract 2

ACC1 negatively regulates Treg activity. ACC1 inhibition augments Treg suppressive and metabolic function by enhancing mitochondrial function and fusion.

## Introduction

Regulatory T cells (Tregs) play an important role in maintaining immune homeostasis, and are critical for preventing autoimmune and inflammatory disorders (1–3). Treg adoptive transfer is effective in preventing murine graft-versus-host disease (GVHD), a major cause of patient morbidity and mortality after allogeneic hematopoietic stem cell transplantation (4, 5), by limiting alloimmune responses (6, 7). However, clinical translation of Treg adoptive transfer has been challenging, primarily because of difficulties with generating large numbers of Tregs with full suppressive capacity (8–11). Despite these limitations, both phase I and II clinical trials using prophylactic Treg adoptive transfer have demonstrated reduced acute and chronic GVHD incidence compared with historical controls (12–17), and a recently completed randomized, phase III trial similarly showed significantly reduced rates of GVHD in patients given Tregs after transplant (18). Another early-phase study demonstrated potential efficacy when using Tregs to treat patients with active GVHD (19). Thus, Tregs are a potentially promising therapy for inflammatory disorders. To obviate the need to produce large numbers of Tregs, strategies to augment Treg suppressive capacity may enhance the translational potential of Treg therapies.

Treg metabolism is tightly linked with suppressor function, and Treg suppressive potency strongly correlates with mitochondrial oxidative phosphorylation (OXPHOS) (20, 21). Upon activation, Tregs can utilize fatty acids (FAs) to drive fatty acid oxidation (FAO) and OXPHOS, which support their survival and function, including expression of suppressive molecules such as cytotoxic T lymphocyte antigen-4 (CTLA-4) and inducible T cell costimulator (ICOS) (20, 22–24). The FA pool that drives FAO can be derived from exogenous FAs, or FAs synthesized by de novo lipogenesis (DNL) (25–27). The balance between FA utilization (FAO) and creation (DNL) is regulated by two isoenzymes, acetyl-CoA carboxylase-1 and -2 (ACC1 and ACC2), both of which convert acetyl-CoA to malonyl-CoA, although in separate subcellular locations (25–27). ACC1 localizes to the cytoplasm, and is thought to initiate DNL through cytosolic malonyl-CoA production (26, 27). In contrast, ACC2 localizes to the outer mitochondrial membrane, where it produces malonyl-CoA at the mitochondrial surface. In turn, malonyl-CoA competitively inhibits the activity of carnitine palmitoyltransferase 1a (CPT1a), the rate-limiting enzyme in FAO (26–28). Thus, ACC1 and ACC2 work cooperatively, but independently to initiate DNL and inhibit FAO.

In naive CD4^+^ conventional T cells (Tcons), inhibition or genetic deletion of ACC1 increases FA uptake and reduces Tcon glycolysis (29, 30). This metabolic shifting enhances naive CD4^+^ Tcon differentiation in vitro to induced Tregs (iTregs), in part because of enhanced acetyl-CoA levels, which drive epigenetic changes that increase Foxp3 expression (29, 31). Tcons from ACC1-knockout (ACC1KO) mice fail to induce murine GVHD, partly because of increased peripheral Treg numbers in recipient tissues (30). In the tumor microenvironment, ACC1 is found at high levels in CD8^+^ Tcons, and inhibiting ACC1 augments FA utilization and FAO and functionally reinvigorates CD8^+^ Tcons against tumors (32). Thus, modulating ACC1 in Tcons has a marked impact on their differentiation and functionality. However, despite the seemingly important relationship between FAs and FAO in Tregs, a Treg-intrinsic role for ACC1 has not yet been evaluated.

Here we show that pharmacologic inhibition or genetic deletion of ACC1 significantly increases murine Treg suppressive function in vitro, and, as compared with wild-type (WT), ACC1KO Tregs had an enhanced ability to treat and prevent murine GVHD. Mechanistically, enhanced Treg function with ACC1 inhibition or gene deletion was dependent on FAO-mediated augmentations in OXPHOS, mitochondrial function, and mitochondrial fusion. Conversely, inhibition of mitochondrial fusion abrogated beneficial functional and metabolic effects of ACC1 inhibition. Directly promoting mitochondrial fusion, even in the absence of ACC1 modulation, resulted in a functional and metabolic phenotype similar to ACC1 inhibition, both in vitro and in vivo. Finally, ex vivo–expanded human Tregs incubated with a clinically relevant ACC1 inhibitor resulted in similar in vitro and in vivo functional augmentation to murine Tregs, suggesting that ACC1 manipulations may be a viable strategy to enhance Treg function in patients.

## Results

### ACC1 inhibition increases Treg suppressive function and alters Treg suppressive phenotype.

**Figure 1:**
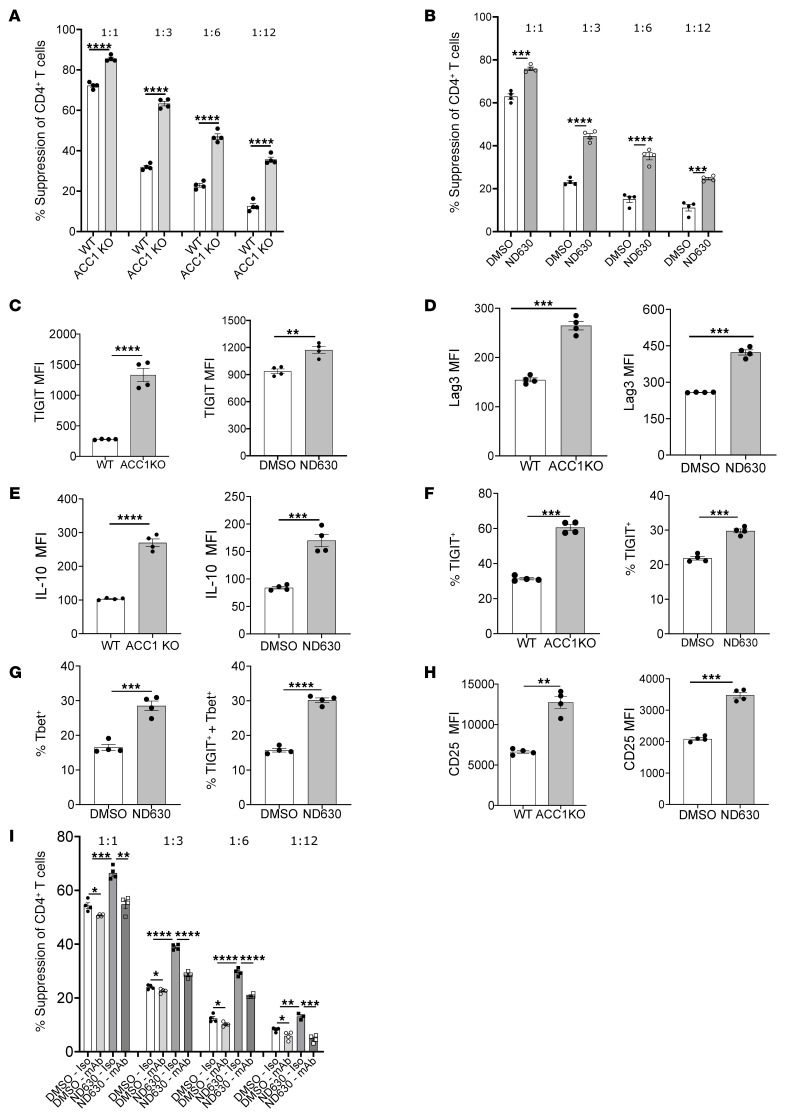
ACC1 inhibition enhances Treg suppressive function and suppressive molecule expression. (**A** and **B**) Suppression of CD4^+^ Tcon proliferation in in vitro Treg suppression assays by WT versus ACC1KO Tregs (**A**) and DMSO- versus ND630-pretreated Tregs (**B**); 1:1–1:12 Treg/Tcon ratios are denoted. (**C**–**H**) Flow cytometry of suppressive molecule expression on overnight-activated WT versus ACC1KO or DMSO- versus ND630-pretreated Tregs. (**C**–**E**) Expression of TIGIT (**C**), Lag3 (**D**), and IL-10 (**E**) on Tregs, as evaluated by median fluorescence intensity (MFI). (**F** and **G**) Frequency of TIGIT^+^ (**F**), T-bet^+^, and TIGIT^+^T-bet^+^ double-positive (**G**) Tregs within the CD4^+^CD25^+^Foxp3^+^ population. (**H**) Expression of CD25 on CD4^+^Foxp3^+^ cells. (**I**) Suppression of CD4^+^ Tcon proliferation in in vitro suppression assays by DMSO- versus ND630-pretreated Tregs, with cultures including either isotype antibodies or a combination of blocking antibodies against Lag3, TIGIT, IL-10, and IL-10 receptor. Data show 1 experiment representative of 3 independent experiments with *n* = 4 replicates per group. *P < 0.05, **P < 0.01, ***P < 0.001, and ****P < 0.0001 by unpaired *t* test or 1-way ANOVA. Error bars represent the mean ± SEM.

To assess the Treg-intrinsic role of ACC1 in Treg suppressive function, we first generated Treg-specific ACC1-deficient mice (ACC1^fl/fl^ × FoxP3-YFP-Cre; ACC1KO). ACC1KO mice did not develop any signs of autoimmunity and, compared with WT mice, demonstrated similar percentages of splenic Tregs (Supplemental Figure 1A; supplemental material available online with this article; https://doi.org/10.1172/JCI182480DS1), with similar distributions of naive and memory-like populations of splenic CD4^+^ and CD8^+^ Tcons (Supplemental Figure 1, B and C). Next, we evaluated the functional capacity of ACC1KO Tregs compared with WT Tregs in standard in vitro suppression assays. Compared with controls, ACC1KO Tregs demonstrated significantly higher suppression of both CD4^+^ and CD8^+^ Tcon proliferation (Figure 1A and Supplemental Figure 2A). To evaluate a translationally relevant approach for ACC1 inhibition, we assessed the effect of pharmacologic ACC1 inhibition on WT Treg function using the clinically relevant ACC1 inhibitor ND630 (33). As compared with control Tregs, a 2-hour pretreatment with ND630, followed by multiple washes to remove excess inhibitor, enhanced in vitro Treg suppression, as seen with ACC1KO Tregs (Figure 1B and Supplemental Figure 2B). Notably, pretreatment of WT Tregs with either of two other ACC inhibitors, TOFA (RMI14514) and Soraphen A, each of which has higher specificity for ACC1 than ND630 but are less translationally relevant, similarly increased Treg suppressive function (Supplemental Figure 2C). Pretreatment of ACC1KO Tregs with TOFA or Soraphen A had no additive effect on suppression (Supplemental Figure 2D), suggesting there were no beneficial off-target effects of these drugs.

Although ACC1 and ACC2 are generally thought to work in a complementary fashion to promote DNL (ACC1) and inhibit FAO (ACC2) (27, 34), neither a Treg-specific knockout of ACC2 (ACC2^fl/fl^ × FoxP3-YFP-Cre) nor pretreatment of WT Tregs with an ACC2-specific pharmacologic inhibitor (CD-017-0191) enhanced Treg function in vitro (Supplemental Figure 2, E and F). This is consistent with previous data in Tcons, which have roughly 10-fold higher relative expression levels of ACC2 compared with Tregs (Supplemental Figure 2F), showing no functional effect of ACC2 modulation (29).

To begin to elucidate the mechanism by which ACC1 inhibition augments Treg function, we characterized Treg suppressive molecule expression after in vitro activation. Compared with controls, both ACC1KO and ND630-treated Tregs showed increased expression of the immunosuppressive molecules T cell immunoreceptor with immunoglobulin and ITIM domain (TIGIT) and lymphocyte activation gene-3 (LAG3) and the immunosuppressive cytokine IL-10, and an overall higher proportion of TIGIT^+^ Tregs (Figure 1, C–F). The higher proportion of TIGIT^+^ Tregs also corresponded to an increased proportion of Tregs expressing the transcription factor T-box in T cells (T-bet) (Figure 1G, left), as well as an increased frequency of TIGIT^+^T-bet^+^ double-positive Tregs (Figure 1G, right). In addition to suppressive molecules, ACC1KO and ND630-treated Tregs each expressed higher levels of CD25 after activation (Figure 1H), but there were no differences in expression of Foxp3, CTLA-4, or programmed cell death protein-1 (PD-1) (Supplemental Figure 3, A and B). When added separately to Treg suppression assays, blocking antibodies against TIGIT, LAG3, or IL-10/IL-10 receptor did not abrogate the beneficial effect of ND630 (Supplemental Figure 3, C–E). However, a combination of blocking antibodies against TIGIT, LAG3, and IL-10/IL-10 receptor significantly diminished the suppressor function of ND630-treated Tregs to a greater extent than seen in controls (Figure 1I and Supplemental Figure 3F), suggesting a combinatorial effect of the upregulated molecules in augmenting Treg suppression in vitro.

**Figure 2:**
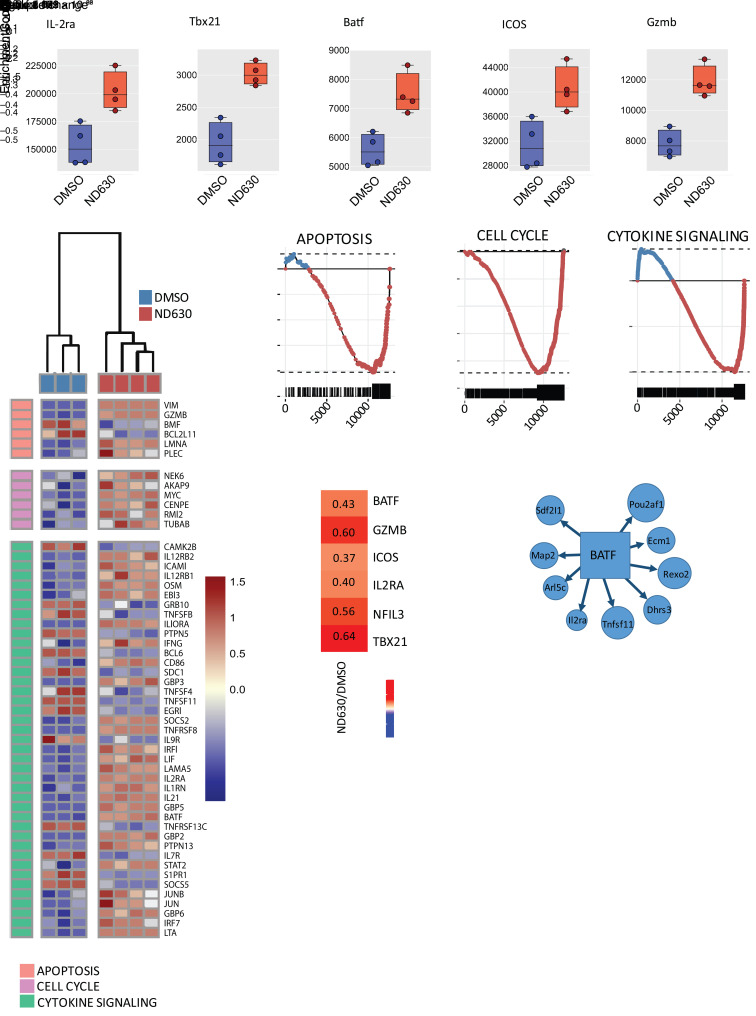
ACC1 inhibition alters Treg expression patterns. (**A**–**E**) Bulk RNA sequencing results of overnight-activated Tregs pretreated with either DMSO or ND630. (**A**) Pairwise comparisons of selected differentially regulated genes between DMSO-pretreated (blue) and ND630-pretreated (red) Tregs, including gene expression of *IL2ra* (CD25), *Tbx21* (T-bet), *Batf* (BATF), *Icos* (ICOS), and *Gzmb* (GZMB). (**B**) Heatmap of differentially enriched genes as clustered by Reactome gene set enrichment analysis (GSEA) nodes. DMSO samples are indicated at the top in blue, whereas ND630 samples are in red. (**C**) Individual enrichment barcode plots for the Reactome GSEA nodes shown in **B**. Genes enriched in ND630-treated Tregs are below the axis (red); genes enriched in DMSO-treated Tregs are above (blue). (**D**) Heatmap showing the relative gene fold change between ND630- and DMSO-treated Tregs. (**E**) Gene-regulatory network analysis (GRNA) highlighting a regulatory module with BATF as the primary node. Three (DMSO) or four (ND630) individual samples were collected and analyzed. Differentially expressed genes were defined as having an adjusted *P* value of less than 0.05 and a log_2_ fold change greater than 0.15.

Unbiased bulk RNA sequencing of overnight-activated Tregs pretreated with DMSO or ND630 revealed a distinct gene expression profile after ACC1 inhibition. Principal component analysis indicated independent clustering of DMSO and ND630 samples, with approximately 80% of variance in gene expression between groups attributed to ND630 treatment (Supplemental Figure 4A). In total, 399 genes were differentially expressed between control and ND630-treated Tregs, 178 of which were upregulated after ND630 treatment. Consistent with CD25 and T-bet protein expression by flow cytometry, expression of the genes encoding CD25 (*Il2ra*) and T-bet (*Tbx21*) was increased after ND630 treatment (Figure 2, A, B, and D), and *Tbx21* was among the 100 most differentially expressed genes (Supplemental Figure 4B). We also noted a significant increase in expression of the genes encoding basic leucine zipper transcription factor (BATF), which has been associated with a highly suppressive “effector” Treg phenotype (35), and ICOS and granzyme B (Figure 2, A and D), similarly implicated in Treg suppressive function (36, 37). Reactome gene set enrichment analysis (38) further highlighted upregulation of BATF, ICOS, and granzyme B (Figure 2B), and an overall skewing toward Treg activation (Figure 2, B and C). Gene regulatory network analysis (GRNA) (39) of the differentially expressed genes between DMSO and ND630 groups further identified BATF as a major coregulatory node in conjunction with nuclear factor interleukin 3 (Nfil3), a known BATF transcriptional regulation partner in Tregs (40) with increased expression after ND630 treatment (Figure 2D), with GRNA predicting CD25 to be under regulatory control of BATF/Nfil3 (Figure 2E). Overall, these data show that ACC1 deletion or pharmacologic inhibition enhances Treg suppressive capacity, augments expression of known suppressive molecules, and skews Tregs toward an “effector” phenotype.

### ACC1 deletion augments Treg-mediated prevention of murine acute GVHD.

**Figure 3:**
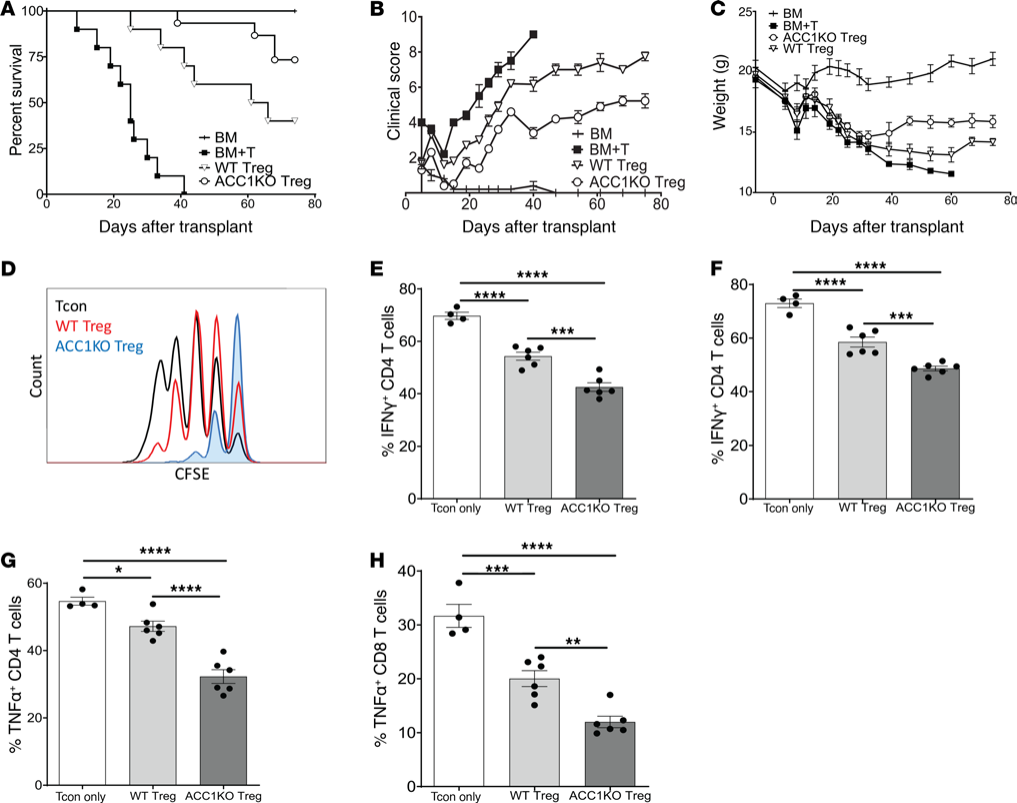
ACC1 inhibition enhances Treg-mediated prevention of acute GVHD. (**A**–**C**) Acute GVHD transplant with BALB/c recipient mice given BM, BM plus T cells (BM+T), or BM+T with either WT Tregs or ACC1KO Tregs. Survival (**A**), clinical scores (0, no disease; 10, severe disease) (**B**), and weights (**C**) of recipient mice after transplant. Data are representative of 2 independent experiments, with *n* = 10 BM only, *n* = 10 acute GVHD, *n* = 10 WT Tregs, and *n* = 12 ACC1KO Tregs. (**D**–**H**) Lethally irradiated BALB/c recipients given CSFE-labeled Tcons alone (Tcon) or CFSE-labeled Tcons with either WT or ACC1KO Tregs. After 72 hours, splenocytes were harvested for analysis. (**D**) CFSE analysis of splenic donor CD8^+^ Tcon proliferation. (**E** and **F**) IFN-γ expression in splenic donor CD4^+^ and CD8^+^ Tcons after ex vivo restimulation. (**G** and **H**) TNF-α expression in splenic donor CD4^+^ and CD8^+^ Tcons after ex vivo restimulation. Data represent 2 independent experiments; *n* = 4 for Tcons only, *n* = 6 for WT Tregs, and *n* = 6 for ACC1KO Tregs. *P < 0.05, **P < 0.01, ***P < 0.001, and ****P < 0.0001 by log-rank test (survival) or 1-way ANOVA. Error bars represent the mean ± SEM.

Given the clinical interest in developing and enhancing Treg therapies for GVHD, we evaluated whether Treg-specific ACC1 deletion would increase the ability of Tregs to prevent acute GVHD (aGVHD). BALB/c (H-2^d^) recipient mice were conditioned with total-body irradiation (TBI), then infused with C57BL/6 (B6; H-2^b^) bone marrow (BM) without or with B6 Tcons (CD4^+^CD25^–^ and CD8^+^CD25^–^) (41, 42). To evaluate Treg-mediated prevention of aGVHD, some mice were also given WT or ACC1KO Tregs at the time of transplant (41). Mice given BM and Tcons developed severe systemic inflammation, with rapid death, severe clinical GVHD scores (0, no disease; 10, severe disease), and weight loss (Figure 3, A–C). All mice receiving Tregs demonstrated improved recipient survival, reduced GVHD clinical scores, and improved weights compared with mice receiving BM and Tcons only (Figure 3, A–C), indicating a reduction in GVHD severity. However, compared with recipient mice given WT Tregs, mice infused with ACC1KO Tregs had improved survival, GVHD clinical scores, and weights (Figure 3, A–C), indicating further enhancement in GVHD suppression with ACC1KO Tregs. This improvement in GVHD severity with ACC1KO Tregs was correlated with reduced donor Tcon proliferation early (day 3) after transplant (Figure 3D), and reduced inflammatory cytokine production by donor CD4^+^ and CD8^+^ Tcons on day 14 (D14) after transplantation (Figure 3, E–H). These data demonstrate that Treg-specific ACC1 deletion enhances Treg-mediated prevention of aGVHD.

### ACC1 deletion augments Treg-mediated treatment of established murine chronic GVHD.

**Figure 4:**
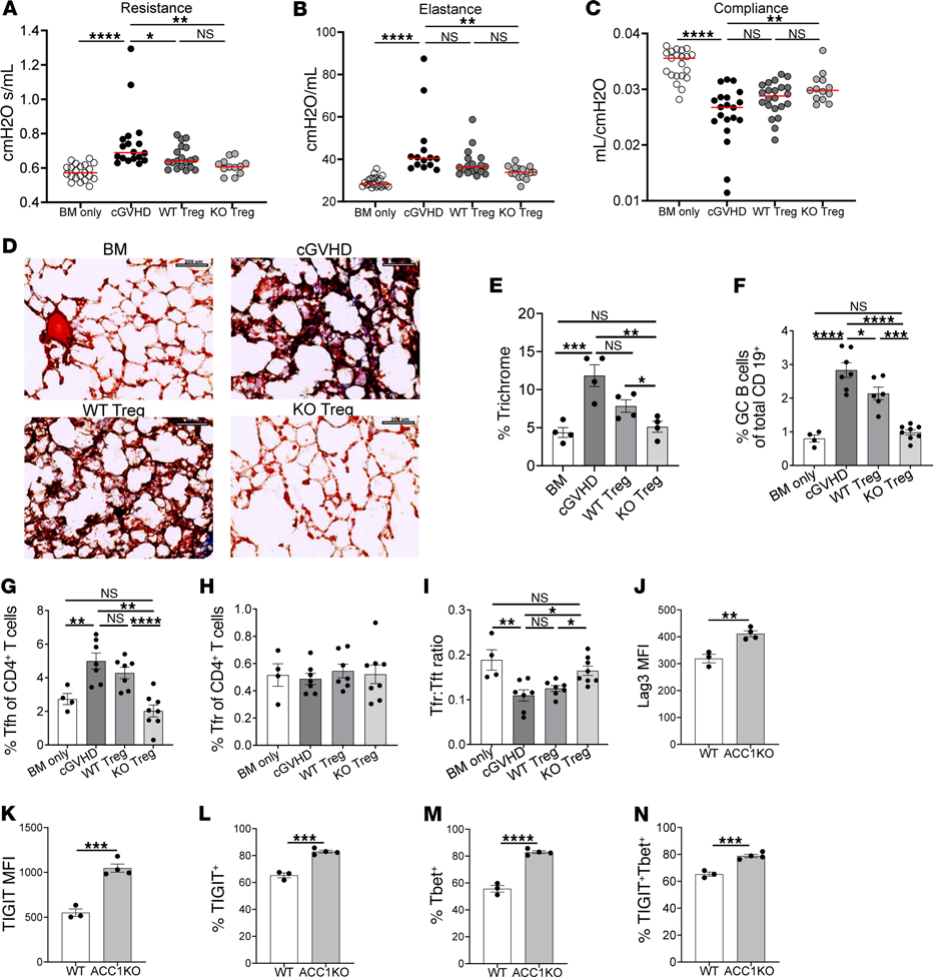
ACC1 inhibition enhances Treg-mediated treatment of chronic GVHD. Chronic GVHD (cGVHD) transplants using B10.BR recipients receiving BM cells (BM) ± B6 T cells (D0). Two groups of mice given BM+T cells were subsequently given WT or ACC1KO (KO) Tregs on D28 after transplant. (**A**–**C**) Pulmonary function tests obtained on D49 after transplant showing airway resistance (**A**), lung elastance (**B**), and total lung compliance (**C**) of mice given either BM alone (BM only), BM+T cells (cGVHD), or BM+T cells with WT Tregs or ACC1KO Tregs. Data are pooled from 3 transplants, with *n* = 22 BM only, *n* = 21 cGVHD, *n* = 20 WT Tregs, and *n* = 15 ACC1KO Tregs. (**D** and **E**) Cryopreserved lung sections obtained on D50 after transplant, stained with Masson’s trichrome, and analyzed for collagen deposition. Scale bars: 200 μm. (**D** and **E**) Representative images of trichrome staining (**D**) and quantification of the trichrome area (**E**), with *n* = 4 images per group. (**F**–**I**) Flow cytometry of splenocytes obtained on D50 after transplant showing the frequency of germinal center B cells (GC B cells; GL7^+^FAS^hi^ of CD19^+^ cells) (**F**), follicular helper T cells (Tfh cells; CXCR5^+^PD-1^+^Foxp3^–^ of CD4^+^ cells) (**G**), and follicular regulatory T cells (Tfr cells; CXCR5^+^PD-1^+^Foxp3^+^ of CD4^+^ cells) (**H**) and the ratio of Tfr to Tfh cells (**I**). Data show 1 experiment representative of 3 independent experiments with *n* = 4 for BM only, *n* = 7 for cGVHD, *n* = 6 for WT Tregs, and *n* = 8 for KO Tregs. (**J**–**N**) Flow cytometry of WT and ACC1KO Tregs on D42 after transplant showing expression of LAG3 (**J**) and TIGIT (**K**), and frequency of TIGIT^+^ (**L**), T-bet^+^ (**M**), and TIGIT^+^T-bet^+^ double-positive (**N**) Tregs within the donor CD4^+^CD25^+^Foxp3^+^ population. *P < 0.05, **P < 0.01, ***P < 0.001, and ****P < 0.0001 by unpaired *t* test or 1-way ANOVA. Error bars represent the mean ± SEM.

We next tested whether Treg-specific ACC1 deletion would increase the ability of Tregs to treat mice with established chronic GVHD (cGVHD). B10.BR (H-2^k^) recipient mice were conditioned with cyclophosphamide and TBI, and then infused with T cell–depleted B6 (H-2^b^) BM without or with B6 T cells (43, 44). Mice given BM and T cells (cGVHD) develop multiorgan systemic fibrosis including pulmonary manifestations (bronchiolitis obliterans), whereas those receiving BM alone serve as no-cGVHD controls. On day 28, cGVHD mice were given either WT or ACC1KO Tregs as therapy for established disease (45). Recipients that received ACC1KO Tregs demonstrated significantly improved lung function parameters compared with mice with cGVHD, whereas mice given WT Tregs showed minimal improvement (Figure 4, A–C). Similarly, lung function of mice given ACC1KO Tregs was similar to that of BM-only (no-cGVHD) controls (Supplemental Figure 5A). Consistent with these data, evaluation of pulmonary fibrosis by trichrome staining demonstrated a reduction in pulmonary fibrosis in mice given ACC1KO Tregs compared with mice with cGVHD and those given WT Tregs, with no difference between BM only and mice given ACC1KO Tregs (Figure 4, D and E).

The germinal center (GC) response, collaborative interactions between GC B cells and CD4^+^ follicular helper T (Tfh) cells, can lead to B cell class switching, and ultimately deposition of high-affinity IgG antibodies in cGVHD target organs, critical drivers of cGVHD pathogenesis in this model (44). We reported previously that infusing Tregs into mice with established cGVHD can abrogate the GC reaction, leading to improved lung function and reduced fibrosis (45). Compared with recipients given WT Tregs, mice given ACC1KO Tregs had a reduced proportion of both GC B cells and Tfh cells (Figure 4, F and G). We also found a significant increase in the ratio of follicular regulatory T (Tfr) cells to Tfh cells in mice receiving ACC1KO Tregs compared with controls, consistent with a more suppressive GC environment (Figure 4, H and I) (46). Taken together, these data highlight that Treg-specific targeting of ACC1 augments the ability of Tregs to treat established cGVHD.

### ACC1 deletion enhances the Treg suppressive phenotype in vivo.

Given our positive in vivo data in 2 different models of GVHD, we next evaluated whether the Treg phenotypic signature observed in vitro after ACC1 deletion could be identified in vivo. We evaluated the phenotype of WT and ACC1KO Tregs infused on D28 after transplant in our cGVHD model, profiling them weekly on D35, D42, and D49 after transplant. The pre-infusion, freshly purified WT and ACC1KO Tregs demonstrated no differences in expression of Foxp3, TIGIT, Lag3, or T-bet (Supplemental Figure 5B), indicating no baseline differences in the infused populations in a naive, non-activated state. There were no differences in expression of Foxp3 over time in vivo (Supplemental Figure 5C), similar to our in vitro results. However, consistent with our in vitro data, ACC1KO Tregs demonstrated increases in Lag3 and TIGIT expression, with higher proportions of TIGIT^+^, T-bet^+^, and TIGIT^+^T-bet^+^ double-positive cells starting at D35 and persisting through D49 (Figure 4, J–N, and Supplemental Figure 5, D and E). These in vivo results indicate that the Treg “effector” signature we identified in vitro after ACC1 inhibition can be identified in vivo, and is persistent over time in the setting of cGVHD.

### Treg mitochondrial metabolism is augmented by ACC1 deletion or pharmacologic inhibition.

**Figure 5:**
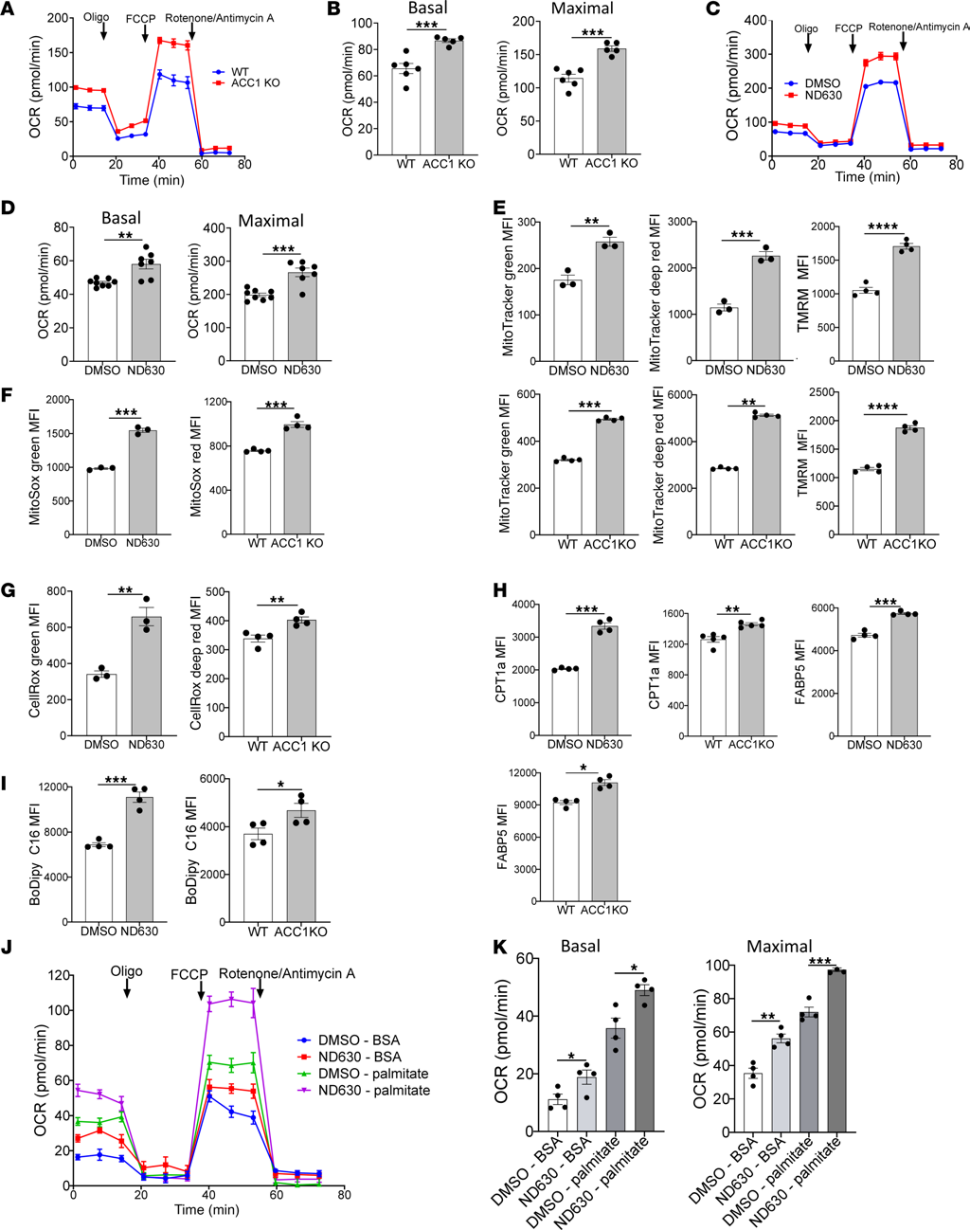
ACC1 inhibition enhances Treg OXPHOS and exogenous FA utilization. (**A**–**D**) Oxygen consumption rate (OCR) analysis in Seahorse Mito Stress test, comparing overnight-activated WT and ACC1KO Tregs (**A** and **B**) or DMSO- and ND630-pretreated Tregs (**C** and **D**). (**A**) OCR curves of WT versus ACC1KO Tregs in response to oligomycin, FCCP, and rotenone/antimycin A, with quantification (**B**); *n* = 6 per group WT, *n* = 5 per group ACC1KO. (**C**) OCR curves of DMSO- versus ND630-pretreated Tregs, with quantification (**D**); *n* = 8 per group DMSO, *n* = 7 per group ACC1KO. (**E**) Flow cytometry of mitochondrial mass (MitoTracker Green, Deep Red) and mitochondrial polarization (TMRM) comparing DMSO- and ND630-pretreated Tregs (*n* = 3 per group) or WT and ACC1KO Tregs (*n* = 4 per group). (**F** and **G**) Flow cytometry evaluation of ROS in WT versus ACC1KO Tregs or DMSO- versus ND630-pretreated Tregs. (**F**) Mitochondrial superoxide evaluation using either MitoSox Green (DMSO vs. ND630, *n* = 3 per group) or MitoSox Red (WT vs. ACC1KO, *n* = 4 per group). (**G**) Total cellular ROS evaluation using CellRox Green (DMSO vs. ND630) or CellRox Deep Red (WT vs. ACC1KO). (**H**) Flow cytometry of CPT1a and FABP5 expression comparing DMSO and ND630 Tregs (*n* = 4 per group) or WT and ACC1KO Tregs (*n* = 4 per group). (**I**) Flow cytometry of BODIPY C16 uptake comparing DMSO and ND630 Tregs (*n* = 4 per group) or WT and ACC1KO Tregs (*n* = 4 per group). (**J** and **K**) OCR analysis in Seahorse Mito Stress tests comparing DMSO and ND630 Tregs. One hour before the assay, Tregs were resuspended in media with either 300 nM BSA or 300 nM BSA-palmitate conjugate. OCR curves of DMSO- versus ND630-pretreated Tregs (**J**) and quantification of OCR (**K**); *n* = 4 per group. All data show representative experiments of 3 or 4 independent experiments. *P < 0.05, **P < 0.01, ***P < 0.001, and ****P < 0.0001 by unpaired *t* test. Error bars represent the mean ± SEM. MFI, median fluorescence intensity.

With the augmentation in Treg function after ACC1 inhibition, and the link between ACC isoenzymes and FAO, we next evaluated whether ACC1 inhibition would increase Treg metabolic function. In Seahorse mitochondrial stress test assays (Agilent) evaluating OXPHOS, ACC1KO and ND630-treated Tregs each demonstrated significantly higher oxygen consumption rates (OCRs) compared with controls, both at baseline and with maximal respiration (Figure 5, A–D). Seahorse glycolysis stress test assays, which evaluate glucose utilization by measuring extracellular acidification rates, did not reveal a significant difference in glycolysis between control, ACC1KO, and ND630-treated Tregs (Supplemental Figure 6, A–D). ACC1KO Tregs had a slight but significant increase in glycolytic capacity compared with control Tregs (Supplemental Figure 6, A and B), indicating a slightly higher capacity to convert glucose to pyruvate or lactate under mitochondrial stress conditions (47). However, compared with control Tregs, neither ACC1KO nor ND630-treated Tregs demonstrated increased expression of the key murine T cell glucose transporter GLUT-1 or increased uptake of the fluorescent glucose analog 2-NBDG after activation (Supplemental Figure 6, E and F). Taken together, these data indicate that ACC1 inhibition substantially increases OXPHOS, with minimal effect on glycolytic pathways.

Assessment of mitochondrial mass and membrane potential, both increased when mitochondria are maximally functional, revealed that compared with controls, both ND630-treated and ACC1KO Tregs had enhanced mitochondrial mass, as assessed by MitoTracker Green and MitoTracker Deep Red (Thermo Fisher Scientific), as well as increased mitochondrial membrane potential, as assessed by tetramethylrhodamine methyl ester (TMRM) (Figure 5E). Congruent with increased OXPHOS and mitochondrial activity, we observed an expected increase in the production of reactive oxygen species (ROS) (48), specifically, mitochondrial superoxide (MitoSox) and total cellular ROS (CellRox, Thermo Fisher Scientific), in both ND630-treated and ACC1KO Tregs, compared with controls (Figure 5, F and G).

To evaluate the specific contribution of FAs and FAO in driving the observed enhancements in OXPHOS, we next assessed expression of the rate-limiting FAO enzyme CPT1a. Compared with controls, both ND630-treated and ACC1KO Tregs expressed significantly more CPT1a (Figure 5H, left). Upstream of CPT1a, fatty acid–binding proteins (FABPs) shuttle FAs to the mitochondria. FABP5, an isoform highly expressed in T cells and important for Tregs (49), was upregulated in both ND630-treated and ACC1KO Tregs compared with controls (Figure 5H, right). Concurrent with these increases in FAO machinery, ND630-treated and ACC1KO Tregs both demonstrated increased uptake of the fluorescent long-chain fatty acid BODIPY C16 (Figure 5I), without a difference in staining for endogenous neutral lipids (BODIPY 493/503) (Supplemental Figure 6G). To determine whether exogenous FAs were drivers of augmented OXPHOS after ACC1 inhibition, we evaluated whether a short (1-hour) pretreatment of control and ND630-treated Tregs with the 16-carbon FA palmitate would affect OCR in Seahorse mitochondrial stress test assays. Consistent with the CPT1a, FABP5, and BODIPY C16 data, ACC1-inhibited Tregs demonstrated an augmented ability to increase OXPHOS in response to palmitate, increasing both basal and maximal OCR (Figure 5, J and K). Overall, these data demonstrate that ACC1 inhibition augments Treg OXPHOS, and drives increases in mitochondrial mass/membrane potential, exogenous FA uptake, and key components of FAO pathways.

### FAO and OXPHOS are required for augmentations in Treg suppression after ACC1 inhibition.

**Figure 6:**
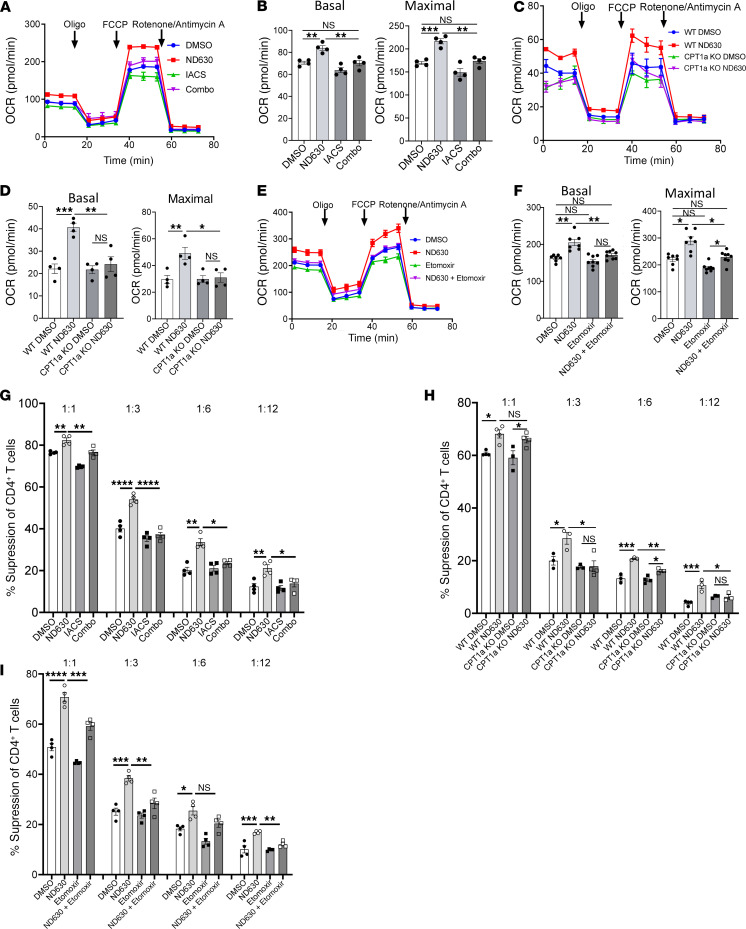
Inhibition of OXPHOS or FAO diminishes the beneficial effect of ACC1 inhibition on Treg function. (**A**–**F**) OCR analysis in Seahorse Mito Stress test. (**A**) OCR curves of Tregs pretreated with DMSO, ND630, IACS, or ND630 plus IACS (Combo), with quantification (**B**); *n* = 4 per group. (**C**) OCR curves of WT and CPT1aKO Tregs pretreated with DMSO or ND630, with quantification (**D**); *n* = 4 per group. (**E**) OCR curves of Tregs pretreated with DMSO, ND630, etomoxir, or ND630 plus etomoxir, with quantification (**F**); *n* = 7 per group for DMSO and ND630, *n* = 8 per group for etomoxir and ND630 plus etomoxir. (**G**–**I**) Suppression of CD4^+^ Tcon proliferation in in vitro suppression assays by Tregs pretreated with DMSO, ND630, IACS, or ND630 plus IACS (Combo) (**G**), WT or CPT1aKO Tregs pretreated with DMSO or ND630 (**H**), and Tregs pretreated with DMSO, ND630, etomoxir, or ND630 plus etomoxir (**I**); 1:1–1:12 Treg/Tcon ratios; *n* = 4 per group, except for **H**, in which some groups have *n* = 3. All data are from representative experiments of 3 independent experiments. *P < 0.05, **P < 0.01, ***P < 0.001, and ****P < 0.0001 by unpaired *t* test or 1-way ANOVA. Error bars represent the mean ± SEM.

We next assessed whether the increased OXPHOS and FAO-related molecules observed with ACC1 inhibition were obligatory for Treg functional enhancements. To evaluate the importance of OXPHOS after ACC1 inhibition, we titrated the mitochondrial electron transport chain complex I inhibitor IACS-010759 (IACS) to the lowest possible dose that resulted in modest but significant and reproducible reductions in Treg OXPHOS (Figure 6, A and B). When Tregs were cotreated with ND630 and IACS, the benefits of ND630 for both basal and maximal OCR were nullified, with OCR levels of cotreated Tregs largely similar to those of control Tregs, and significantly lower than those with ND630 treatment alone (Figure 6, A and B). Combined pretreatment with ND630 and IACS also significantly diminished the functional benefit of ND630, as evidenced by reduced in vitro Treg suppressor function to levels nearly identical to those of controls (Figure 6G and Supplemental Figure 7A).

To interrogate the roles of CPT1a and FAO in augmented Treg suppressor function after ACC1 inhibition, CPT1aKO mice (CPT1a^fl/fl^ × CD4-Cre) were used as a Treg source. CPT1aKO and WT Tregs had similar basal and maximal OCR levels in mitochondrial stress test assays (Figure 6, C and D), and similar in vitro suppressive function (Figure 6H and Supplemental Figure 7B) as previously published (50). However, whereas WT Tregs robustly increased their OCR and suppressive function when treated with ND630, ND630 treatment of CPT1aKO Tregs had virtually no beneficial effect on either OXPHOS or suppressive function (Figure 6, C, D, and H, and Supplemental Figure 7B), pointing to CPT1a and FAO as essential for enhanced Treg function with ACC1 inhibition. To ensure that our results with CPT1aKO Tregs were not solely due to compensatory pathway changes due to genetic knockout, we also used treatment with low-dose etomoxir, a small-molecule CPT1a inhibitor, taking care not to exceed 5 μM treatment, as etomoxir has off-target effects most readily demonstrated at concentrations greater than 5 μM (50). Combined pretreatment of WT Tregs with ND630 and 5 μM etomoxir abrogated the beneficial effects of ND630 on OXPHOS and suppression (Figure 6, E, F, and I, and Supplemental Figure 7C). Together, these results indicate that the Treg functional augmentation after ACC1 inhibition depends on FAO and OXPHOS.

### ACC1 pharmacologic inhibition induces mitochondrial fusion.

Mitochondria continuously undergo a dynamic process of fusion and fission, depending on cellular energy demands (51). Increased mitochondrial fusion elongates mitochondria, bringing cristae into closer contact and enhancing electron transport chain supercomplex formation, thereby facilitating more efficient OXPHOS (52–54). As mitochondria are central to the metabolic and functional changes observed with ACC1 inhibition (Figure 5, A–E, and Figure 6, A and B), we sought to understand whether the increase in OXPHOS driven by ACC1 inhibition might be due to increased mitochondrial fusion. Three complementary high-resolution imaging modalities were used to evaluate alterations in mitochondrial fusion: 3D structured illumination microscopy (SIM), expansion microscopy, and transmission electron microscopy. For experiments using 3D SIM and expansion microscopy, Tregs were purified from mice expressing mitochondrial Dendra2, a photoconvertible green to red fluorescent protein engineered to localize to mitochondria (51).

**Figure 7:**
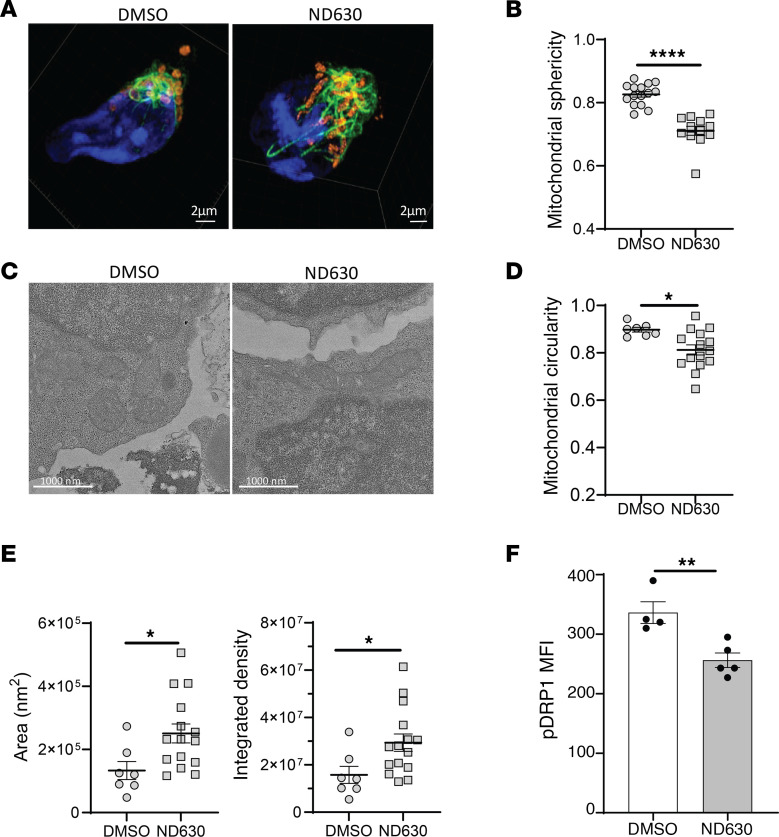
ACC1 inhibition enhances mitochondrial fusion and elongation. (**A** and **B**) 3D SIM imaging of activated Tregs isolated from Dendra2-transgenic mice pretreated with DMSO or ND630. (**A**) Representative images of DMSO- and ND630-pretreated Tregs with DAPI (blue), vimentin (green), and mitochondria (orange). Scale bars: 2 μm. (**B**) Quantification of mitochondrial sphericity per cell from 3D SIM images; *n* = 15 cells in DMSO, *n* = 12 cells in ND630. (**C** and **E**) Electron microscopy imaging of overnight-activated DMSO- and ND630-pretreated Tregs. (**C**) Representative images from DMSO- and ND630-pretreated Tregs depicting intracellular structures, including mitochondrial (roughly centered in each image). Scale bars: 1,000 nm. (**D** and **E**) Quantification of mitochondrial circularity (**D**), mitochondrial area, and mitochondrial integrated density per cell (**E**) from electron microscopy images; *n* = 7 cells in DMSO, *n* = 15 cells in ND630. (**F**) Phosphoprotein flow cytometry of DRP1 comparing DMSO- versus ND630-pretreated Tregs; *n* = 4 DMSO, *n* = 5 ND630. All data are from representative experiments of 2 (electron microscopy) or 3 independent experiments. *P < 0.05, **P < 0.01, ****P < 0.0001 by unpaired *t* test. Error bars represent the mean ± SEM.

Visualized mitochondria from ND630-treated Tregs demonstrated a higher degree of mitochondrial fusion and elongation compared with control Tregs, which were more spherical/circular (Figure 7, A and C, and Supplemental Figure 7D). Quantification of elongation and sphericity/circularity from each imaging modality further showed a significant reduction in sphericity/circularity (increased elongation) after ND630 treatment, which was consistent across modalities (Figure 7, B and D, and Supplemental Figure 7, E–H). Quantification of mitochondria visualized by electron microscopy showed that ND630 treatment resulted in an increase in mitochondrial area and integrated density (Figure 7E and Supplemental Figure 7I), which corresponds to increased mitochondrial fusion/elongation and cristae density (55). Activation-induced phosphorylation of dynamin-related protein-1 (phospho-DRP1) increases mitochondrial fission, thereby reducing mitochondrial fusion and diminishing OXPHOS (56, 57). Consistent with the increased mitochondrial fusion identified by microscopy, ND630 treatment resulted in a corresponding significant decrease in phospho-DRP1 expression by flow cytometry (Figure 7F), further indicating increased mitochondrial fusion potential. Overall, these data demonstrate that ACC1 inhibition increases mitochondrial fusion and elongation.

### Inhibition of mitochondrial fusion diminishes the beneficial effects of ACC1 inhibition.

**Figure 8:**
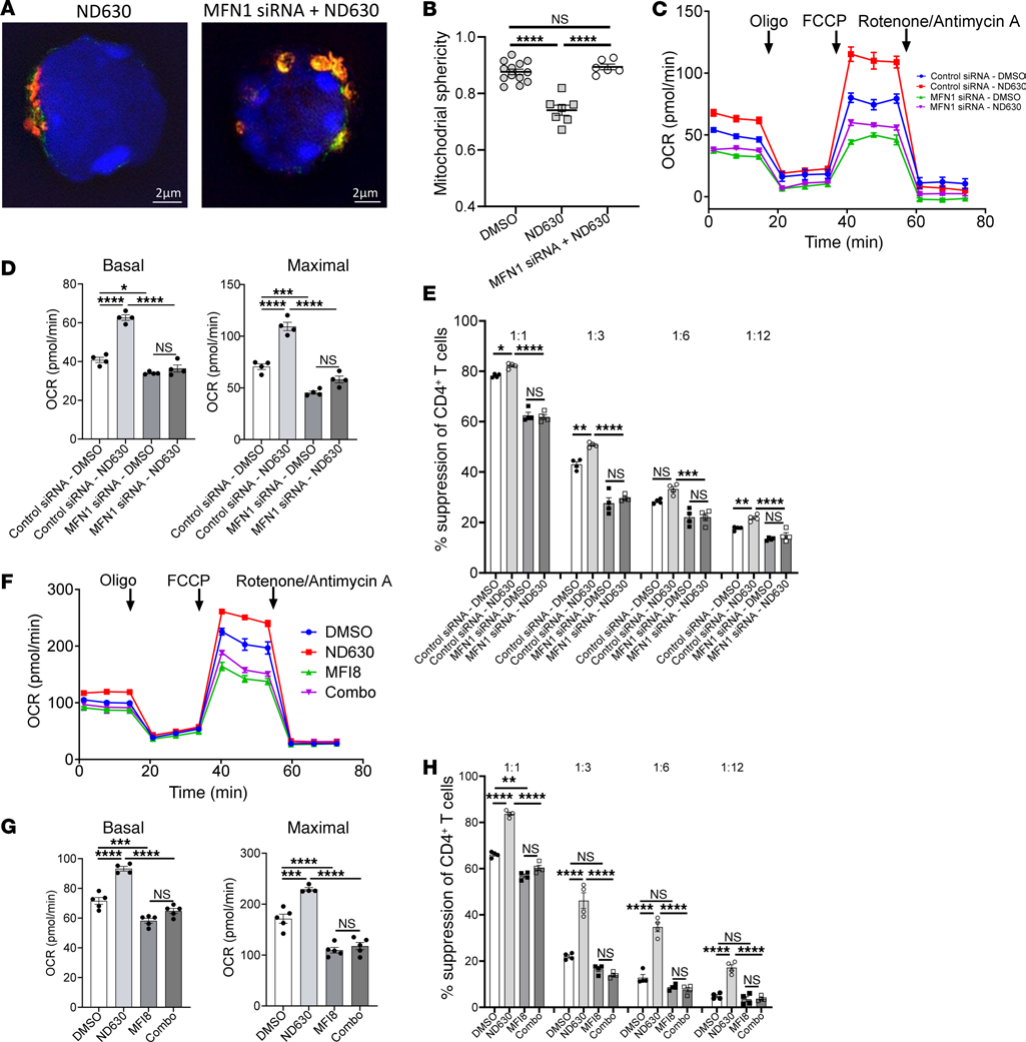
Inhibition of mitochondrial fusion abrogates the beneficial effects of FA synthesis inhibition on Treg function. (**A** and **B**) 3D SIM imaging of control siRNA– or MFN1 siRNA–transduced Dendra2 Tregs pretreated with ND630. (**A**) Representative images of control siRNA– and MFN1 siRNA–transduced Tregs treated with ND630 with staining of DAPI (blue), vimentin (green), and mitochondria (orange). Scale bars: 2 μm. (**B**) Quantification of mitochondrial sphericity per cell from 3D SIM images; *n* = 13 cells in control siRNA+DMSO (DMSO) group, *n* = 7 cells in control siRNA+ND630 (ND630) group, and *n* = 6 cells in MFN1 siRNA+ND630 group. (**C** and **D**) OCR curves of control siRNA and MFN1 siRNA Tregs pretreated with either DMSO or ND630 (**C**), with quantification (**D**); *n* = 4 per group. (**E**) Suppression of CD4^+^ Tcon proliferation in in vitro suppression assays by control siRNA– and MFN1 siRNA–transduced Tregs pretreated with DMSO or ND630; 1:1–1:12 Treg/Tcon ratios; *n* = 4 per group. (**F** and **G**) OCR curves of Tregs pretreated with DMSO, ND630, MFI8, or ND630 plus MFI8 (Combo) (**F**), with quantification (**G**); *n* = 5 per group. (**H**) Suppression of CD4^+^ Tcon proliferation in in vitro suppression assays by Tregs pretreated with DMSO, ND630, MFI8, or ND630 plus MFI8 (Combo); *n* = 4 per group. All data are from representative experiments of 2 (3D SIM) or 3 independent experiments. *P < 0.05, **P < 0.01, ***P < 0.001, and ****P < 0.0001 by unpaired *t* test or 1-way ANOVA. Error bars represent the mean ± SEM.

We next evaluated whether increased mitochondrial fusion was critical for the metabolic and suppressive functional benefits observed with ACC1 deletion or pharmacologic inhibition. Mitofusin protein isoforms 1 and 2 (MFN1 and MFN2) mediate outer mitochondrial membrane fusion, and mitochondrial dynamin-like GTPase (OPA1) mediates inner mitochondrial fusion (57, 58). As MFN2 inhibition or knockdown can increase T cell proliferation and reduce endoplasmic reticulum contact (59, 60) and OPA1 knockdown can affect T cell receptor signaling (61), we chose to assess the functional importance of mitochondrial fusion by targeting MFN1, the knockdown of which has not been associated with the same non-mitochondrial effects as MFN2 or OPA1. Targeting MFN1 with siRNA resulted in an approximately 50% reduction in MFN1 protein, with no off-target reduction in MFN2 (Supplemental Figure 8A). MFN1 knockdown diminished the ability of Tregs to form fused mitochondria and assume an elongated shape in response to ACC1 inhibition (Figure 8, A and B, and Supplemental Figure 8B). This corresponded to decreased OXPHOS, as indicated by lower basal and maximal OCR in mitochondrial stress test assays with MFN1-knockdown Tregs (Figure 8, C and D), and reduced in vitro suppressive function (Figure 8E and Supplemental Figure 8C). MFN1 knockdown similarly diminished ND630 effects on Tregs, as ND630 treatment after MNF1 knockdown failed to increase basal or maximal OCR (Figure 8, C and D), and had no beneficial effect on in vitro suppressive function (Figure 8E and Supplemental Figure 8C).

To further evaluate the role of mitochondrial fusion after ACC1 inhibition, and confirm the MFN1 siRNA data, we tested the effect of MFI8, a recently developed small-molecule inhibitor of MFN1 (58). MFI8 was titrated to the lowest possible dose that resulted in significant and reproducible reductions in Treg OXPHOS and in vitro suppression, without fully abrogating Treg suppressor function. As compared with control Tregs, Tregs pretreated for 2 hours with MFI8 demonstrated a slight but significant reduction in basal and maximal OCR, and diminished in vitro suppressive potency (Figure 8, F–H, and Supplemental Figure 8D). Compared with ND630 treatment alone, cotreatment with MFI8 and ND630 significantly diminished the effect of ACC1 inhibition on basal and maximal OCR (Figure 8, F and G), and nearly completely abrogated the beneficial effect of ND630 treatment on Treg suppressive function (Figure 8H and Supplemental Figure 8D). In aggregate, these data demonstrate that mitochondrial fusion is critical for the downstream suppressive and OXPHOS modulations observed after ACC1 inhibition.

### Directly enhancing mitochondrial fusion augments Treg metabolic and suppressive function.

**Figure 9:**
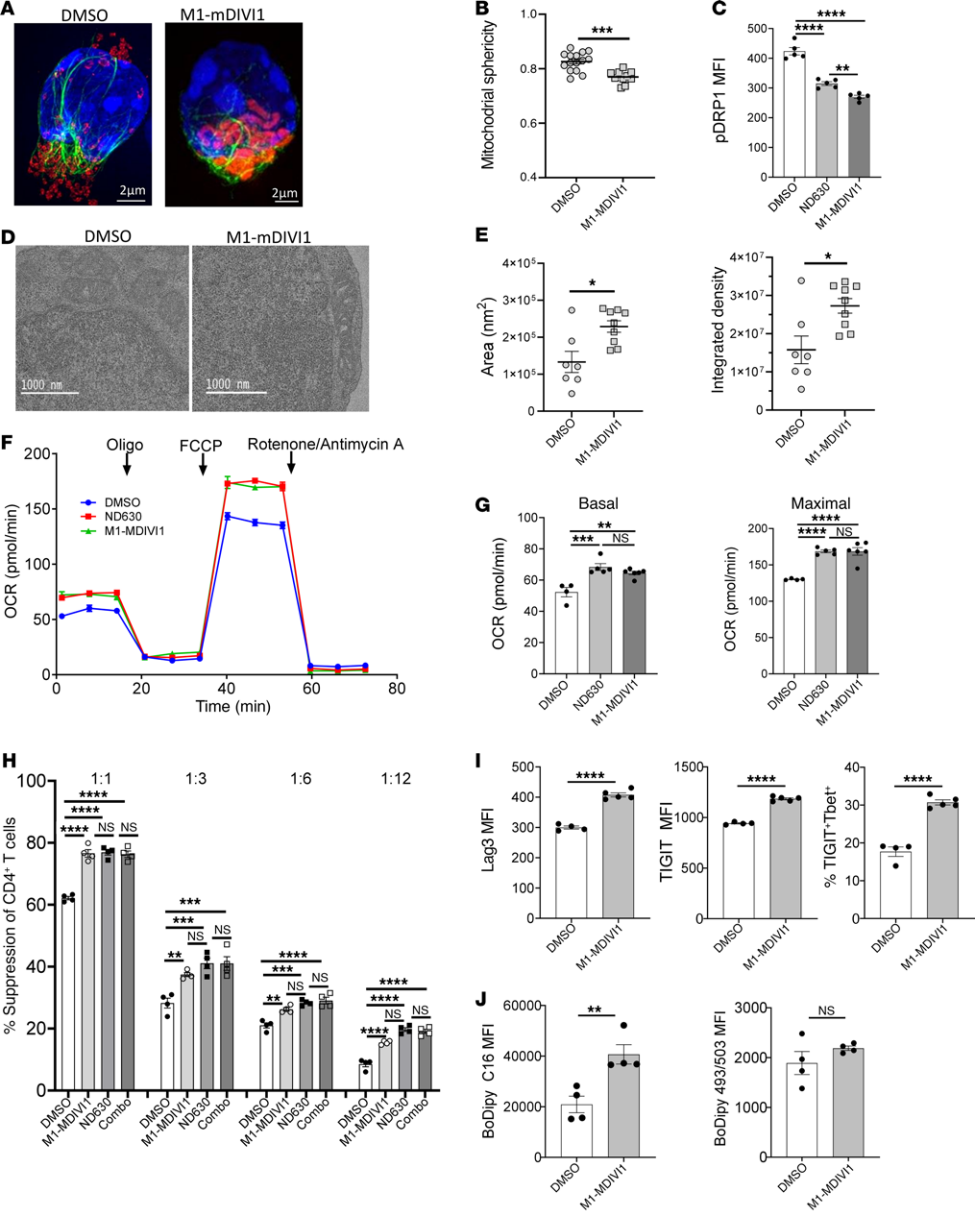
Direct induction of mitochondrial fusion enhances Treg metabolic and suppressive function. (**A** and **B**) 3D SIM imaging of activated Tregs isolated from Dendra2-transgenic mice pretreated with DMSO or M1/MDIVI1. (**A**) Representative images of DMSO- and M1/MDIVI1–pretreated Tregs with staining of nuclei (blue), vimentin (green), and mitochondria (red/orange). Scale bars: 2 μm. (**B**) Quantification of mitochondrial sphericity from 3D SIM images; *n* = 183 DMSO, *n* = 103 M1/MDIVI1. (**C**) Phosphoprotein flow cytometry of phospho-DRP1 comparing DMSO-, ND630-, and M1/MDIVI1–pretreated Tregs; *n* = 4 for DMSO and *n* = 5 for ND630 and M1/MDIVI1 groups. (**D** and **E**) Electron microscopy imaging analysis of overnight-activated DMSO- and M1/MDIVI1–pretreated Tregs. (**D**) Representative images from DMSO- and M1/MDIVI1–pretreated Tregs depicting intracellular structures, including mitochondrial. Scale bars: 1,000 nm. (**E**) Quantification of mitochondrial area and mitochondrial integrated density per cell; *n* = 7 cells in DMSO, *n* = 9 cells in ND630. (**F** and **G**) OCR curves of DMSO-, ND630-, and M1/MDIVI1–pretreated Tregs (**F**), with quantification (**G**); *n* = 4 DMSO, *n* = 5 ND630, *n* = 6 M1/MDIVI1. (**H**) Suppression of CD4^+^ Tcon proliferation in in vitro suppression assays by Tregs pretreated with DMSO, M1/MDIVI1, ND630, or M1/MDIVI1 plus ND630 (Combo); 1:1–1:12 Treg/Tcon ratios; *n* = 4 per group. (**I** and **J**) Flow cytometry comparing overnight-activated DMSO- and M1/MDIVI1–pretreated Tregs. (**I**) Expression of Lag3 and TIGIT on activated Tregs, and frequency of TIGIT^+^T-bet^+^ double-positive Tregs, compared with isotype controls; *n* = 4 per group DMSO, *n* = 5 per group M1/MDIVI1. (**J**) BODIPY C16 uptake (left) and staining of endogenous lipid droplets using BODIPY 493/503 (right); *n* = 4 per group. All data are from representative experiments of 2 (electron microscopy) or 3 independent experiments, with technical replicates shown. *P < 0.05, **P < 0.01, ***P < 0.001, ****P < 0.0001 by unpaired *t* test or 1-way ANOVA. Error bars represent the mean ± SEM.

We next evaluated whether directly augmenting mitochondrial fusion, without ACC1 inhibition, could also enhance Treg functionality. A 2-hour incubation with the combination of the mitochondrial fusion promotor M1 and the mitochondrial fission inhibitor Mdivi-1 (M1/MDIVI1) (54) resulted in a significant increase in mitochondrial fusion by 3D SIM microscopy (Figure 9A), and reduced mitochondrial sphericity (Figure 9B and Supplemental Figure 8F). Flow cytometry revealed a significant reduction in phospho-Drp1, which was diminished to levels below those seen in ND630-treated Tregs (Figure 9C). As with ACC1 inhibition, increased mitochondrial fusion was also observed by electron microscopy (Figure 9D), with a corresponding increase in mitochondrial area and integrated density (Figure 9E and Supplemental Figure 8G). These imaging results confirm induction of mitochondrial fusion by M1/MDIVI1 treatment.

As with ACC1 inhibition, M1/MDIVI1 treatment augmented Treg OXPHOS in comparison with control Tregs, with significant increases in basal and maximal OCR, each comparable to those seen with ND630 treatment (Figure 9, F and G). Moreover, M1/MDIVI1–treated Tregs were superior to control Tregs in their in vitro suppressive function, with suppression nearly comparable to that seen with ND630 treatment (Figure 9H and Supplemental Figure 8E). Cotreatment with M1/MDIVI1 and ND630 was not additive beyond ND630 treatment alone (Figure 9H and Supplemental Figure 8E). M1/MDIVI1 treatment also augmented Treg expression of Lag3 and TIGIT, with a higher frequency of TIGIT^+^T-bet^+^ double-positive Tregs (Figure 9I), and increased uptake of exogenous FAs (BODIPY C16), with no significant change in neutral lipid staining (BODIPY 493/503) in comparison with controls (Figure 9J). Overall, these results suggest that mitochondrial fusion, even in the absence of ACC1 inhibition, can induce metabolic and functional changes that resemble those seen in ACC1-inhibited Tregs.

### Augmenting mitochondrial fusion enhances Treg therapy for established murine cGVHD.

**Figure 10:**
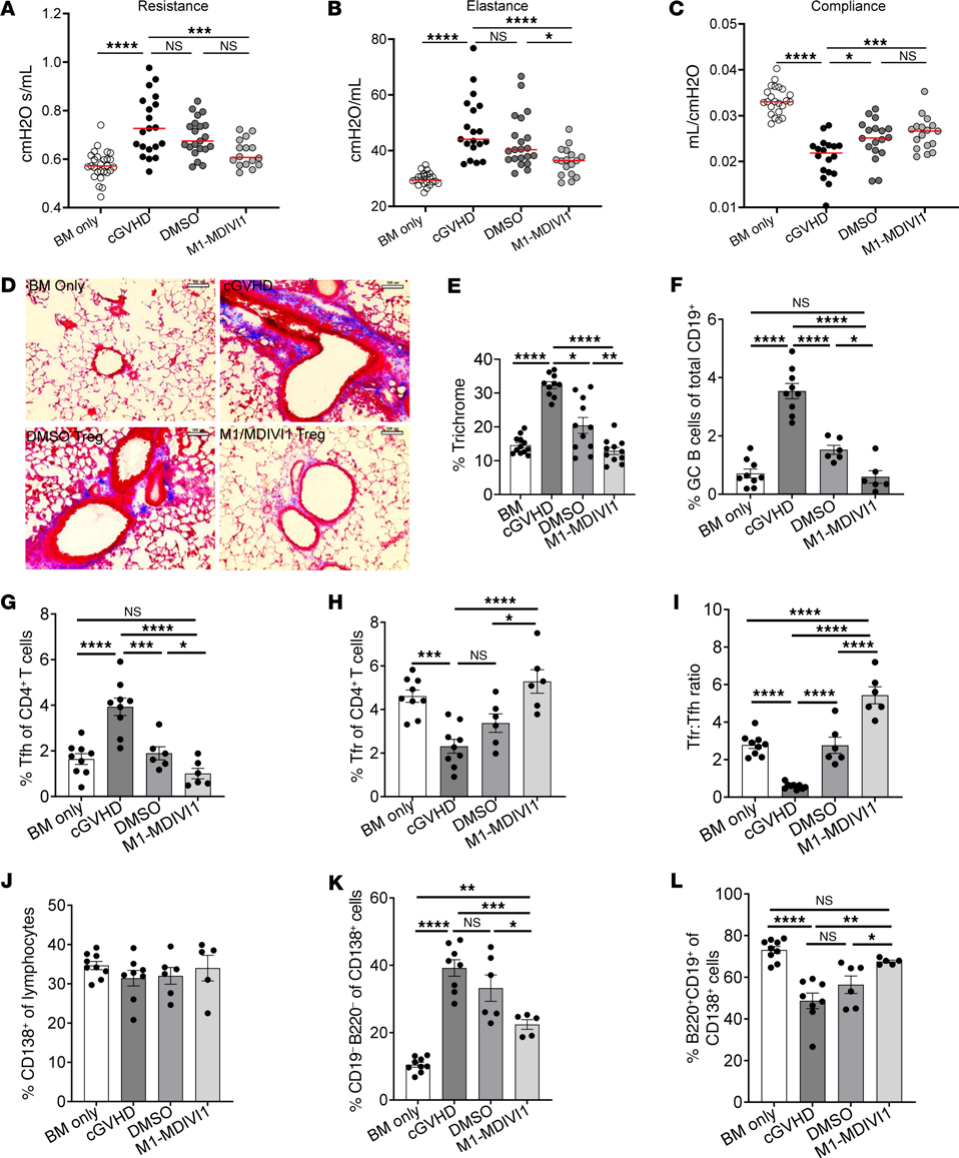
Direct induction of mitochondrial fusion enhances Treg-mediated treatment of cGVHD. Chronic GVHD with B10.BR recipients receiving BM ± B6 T cells (D0). Two groups of mice given BM+T cells were subsequently given DMSO- or M1/MDIVI1–pretreated Tregs on D28 after transplant. (**A**–**C**) Pulmonary function tests obtained on D49 after transplant showing airway resistance (**A**), lung elastance (**B**), and total lung compliance (**C**) of mice given either BM alone, BM+T cells (cGVHD), or BM+T cells and either DMSO- or M1/MDIVI1–pretreated Tregs on D28. Data are pooled from 3 transplants; *n* = 21 BM only, *n* = 18 cGVHD, *n* = 19 DMSO Tregs, *n* = 17 M1/MDIVI1 Tregs. (**D** and **E**) Cryopreserved lung sections obtained on D50 after transplant, stained with Masson’s trichrome, and analyzed for collagen deposition. (**D**) Representative images of trichrome staining (scale bars: 100 μm) and quantification of trichrome area (**E**); *n* = 10 (cGVHD) or *n* = 11 images per group (BM, DMSO, M1/MDIVI1). (**F**–**I**) Flow cytometry of splenocytes obtained on D50 after transplant, with analysis of the frequency of GC B cells (**F**), Tfh cells (**G**), and Tfr cells (**H**) and the ratio of Tfr to Tfh cells (**I**) in recipient mice. Data show 1 experiment representative of 3 independent experiments; *n* = 9 BM, *n* = 9 cGVHD, *n* = 6 DMSO Tregs, *n* = 6 M1/MDIVI1 Tregs. (**J**–**L**) Flow cytometry of lung lymphocytes obtained on D50 after transplant, with analysis of the frequency of total plasma cells (percentage of CD138^+^ cells of live) (**J**), immature plasma cells (% CD19^–^B220^–^ of CD138^+^ cells) (**K**), and mature plasma cells (% CD19^+^B220^+^ of CD138^+^ cells) (**L**) in recipient mice. Data show 1 experiment representative of 3 independent experiments; *n* = 9 BM, *n* = 8 cGVHD, *n* = 6 DMSO Tregs, *n* = 5 M1/MDIVI1 Tregs. *P < 0.05, **P < 0.01, ***P < 0.001, and ****P < 0.0001 by unpaired *t* test or 1-way ANOVA. Error bars represent the mean ± SEM.

We next asked whether directly enhancing mitochondrial fusion with M1/MDIVI1 could augment the ability of Tregs to treat established murine cGVHD. On day 28 after transplant, mice with active cGVHD were given Tregs pretreated with DMSO (control) or M1/MDIVI1. Mice that received M1/MDIVI1–treated Tregs had significantly better lung function compared with mice with cGVHD (Figure 10, A–C), with overall similar pulmonary function to the BM-only control group (Supplemental Figure 8H), as well as reduced lung fibrosis (Figure 10, D and E), fewer GC B cells and Tfh cells (Figure 10, F and G), and an increase in the overall Tfr cell frequency as well as the Tfr/Tfh cell ratio (Figure 10, H and I). In mice receiving M1/MDIVI1 Tregs, GC B cell and Tfh cell frequencies did not significantly differ from those in BM-only mice (Figure 10, F and G), and Tfr cell frequencies actually exceeded those in BM-only mice, increasing the Tfr/Tfh cell ratio above BM-only (Figure 10, H and I).

Flow cytometry evaluation of lung plasma cell populations, including total plasma cells (frequency of CD138^+^ cells), immature plasma cells (CD19^+^B220^+^ of CD138^+^), and mature plasma cells (CD19^–^B220^–^ of CD138^+^), correlates with cGVHD severity, with a higher frequency of mature, antibody-producing plasma cells indicating more severe disease (46). Flow cytometry of lung lymphocytes isolated from the same recipient mice noted above showed similar overall tissue plasma cell frequencies across groups (Figure 10J). However, when compared with mice given control Tregs, mice given M1/MDIVI1–treated Tregs had a reduced frequency of mature (pathogenic) plasma cells (Figure 10K), with a reciprocal increase in the frequency of immature (non-pathogenic) plasma cells (Figure 10L). Together, these results demonstrate that directly inducing mitochondrial fusion in Tregs before adoptive transfer can increase their ability to treat established cGVHD.

### Reduced ACC1 enhances human Treg suppressive function and prevention of xenogeneic GVHD.

**Figure 11:**
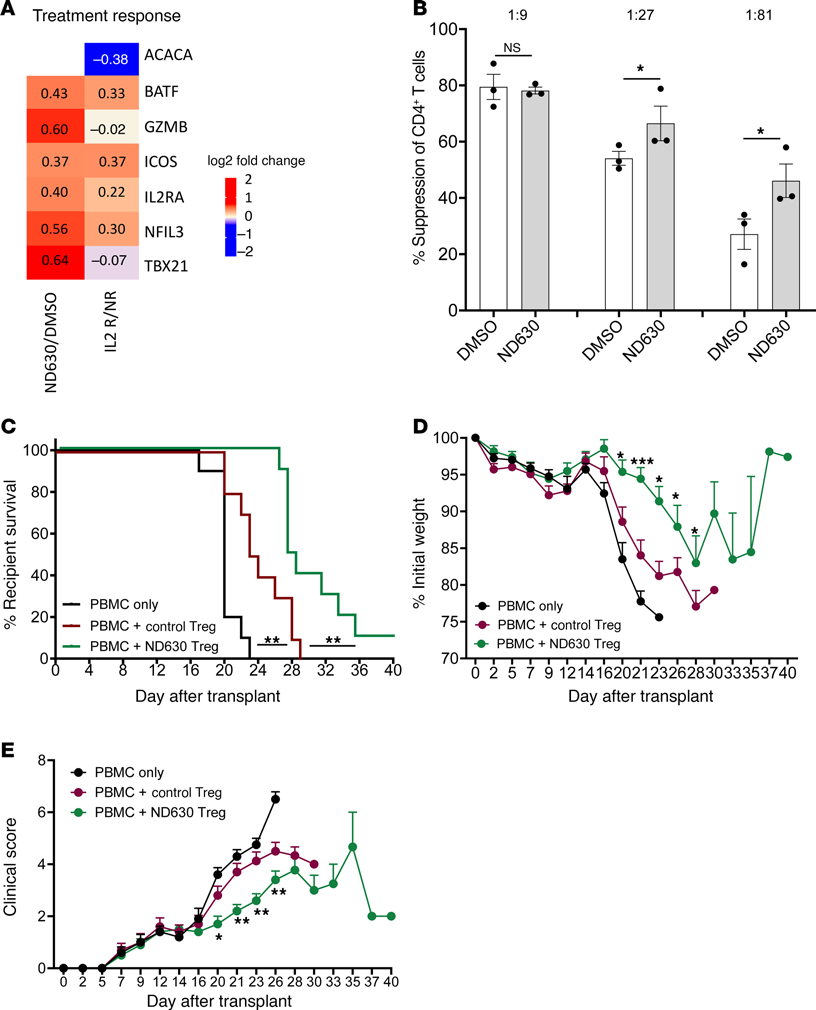
ACC1 inhibition enhances human Treg function in vitro and in vivo. (**A**–**E**) Bulk RNA sequencing results of human Tregs (right column) purified from cGVHD patients who either did (IL-2R) or did not respond to IL-2 therapy (NR) as compared with the bulk RNA sequencing from murine Tregs (left column) treated with ND630 or DMSO, as in Figure 2. (**A**) Heatmap of differentially enriched genes, including those previously evaluated in murine Tregs, showing the relative fold change in gene expression between responders and non-responders; *n* = 6 per group IL-2R, *n* = 5 per group NR. (**B**–**E**) Naive human Tregs were sort-purified, cultured, and expanded, then treated with DMSO or ND630, and then used for functional assays. (**B**) Suppression of CD4^+^ Tcon proliferation in in vitro suppression assays by DMSO- or ND630-pretreated Tregs; 1:9–1:81 Tcon/Treg ratios; *n* = 3 biological replicates per group. (**C**–**E**) Xenogeneic GVHD transplant in which NSG-SGM3 mice were irradiated, then injected with human PBMCs ± DMSO- or ND630-pretreated human Tregs on D0. Assessment of recipient overall survival (**C**), weight loss (**D**), and clinical GVHD score (0, no disease; 10, severe disease) (**E**). Data show 1 experiment representative of 3 independent experiments. *P < 0.05, **P < 0.01, and ***P < 0.001 by unpaired *t* test, and log-rank test for survival analysis (**B**). Error bars represent the mean ± SEM.

To better assess the translational potential of our murine data, we first evaluated whether there were inherent modulations in ACC1 gene expression in human Tregs from patients with cGVHD, and whether we could identify the “effector” Treg gene expression signatures found in murine Tregs in patients who responded to cGVHD therapy. To this end, we performed bulk RNA sequencing on human Tregs isolated from patients with cGVHD treated with low-dose IL-2. In cGVHD, treatment with IL-2 expands and enhances the function of endogenous Tregs, and can improve cGVHD symptoms in patients and nonhuman primates (62–65). Comparisons were made between patients who responded clinically to IL-2 and those who did not. In patients who responded to IL-2 therapy, we noted decreased expression of the gene encoding ACC1 (*Acaca*) in Tregs (Figure 11A), and an overall Treg gene signature that was similar to the “effector” Treg gene expression changes observed after ND630 treatment of murine Tregs (Figure 2, A–E, and Figure 11A). Furthermore, we found at least 22 gene sets with overlapping enrichment between ND630-treated mouse Tregs and human Tregs from patients who responded to IL-2 (Supplemental Figure 9). These data suggest both that ACC1 modulation may be important in human Tregs, and that the “effector” gene expression pattern we identified in mouse Tregs after ACC1 inhibition can be identified in human Tregs, particularly those from cGVHD patients who respond to IL-2 therapy.

We next evaluated whether ACC1 inhibition could augment the function of ex vivo–expanded human Tregs produced identically to those in clinical trials of Treg infusion to prevent acute GVHD (12, 13). Similarly to our murine results, ND630 pretreatment of human Tregs significantly increased in vitro suppression in comparison with controls (Figure 11B). To determine whether ND630-treated human Tregs have increased in vivo potency, a xenogeneic GVHD model was used, in which sublethally irradiated NOD/Scid/IL-2γc^–/–^/IL-3/GM/SF mice were given peripheral blood mononuclear cells (PBMCs) to induce acute xenogeneic GVHD (66, 67). Cohorts also received either no Tregs, control (DMSO) Tregs, or ND630-treated Tregs. Compared with mice receiving PBMCs alone, both groups of mice receiving Tregs had significantly reduced GVHD-induced lethality (Figure 11C) and overall disease severity, as indicated by reduced weight loss and clinical scores (Figure 11, D and E). Consistent with our in vitro results, mice receiving ND630-treated Tregs had significantly increased survival compared with mice receiving control Tregs (Figure 11C), and a further improvement in recipient weights and clinical scores (Figure 11, D and E). Together, these results show that ACC1 inhibition augments human Treg suppressive function in vitro and in vivo in a manner similar to that seen with murine Tregs.

## Discussion

Although Treg therapies for autoimmune and inflammatory disorders have shown promise in the clinic (11–16, 18, 19), effective manipulation of Treg biology to augment Treg therapeutic efficacy remains an unmet need. Here we demonstrate a translationally relevant proof of concept that inhibiting ACC1 enhances Treg functional potency and therapeutic efficacy in two different murine models of GVHD, and in mice with xenogeneic GVHD. Treg-intrinsic ACC1 disruption enhances uptake of exogenous FAs, increases FAO to augment OXPHOS, and enhances mitochondrial fitness and fusion. These metabolic changes are associated with a highly suppressive Treg phenotype, characterized by increased expression of TIGIT, Lag3, IL-10, CD25, ICOS, T-bet, and BATF, among others. Diminishing or blocking FAO, OXPHOS, or mitochondrial fusion abrogates the suppressive benefit of ACC1 inhibition. Thus, the metabolic and mitochondrial changes induced by ACC1 inhibition are critical mediators of the observed downstream suppressive changes. This conclusion is further supported by our finding that bolstering mitochondrial fusion with M1/MDIVI1 augments Treg metabolic and suppressive function, independent of ACC1 inhibition. Finally, these findings also appear applicable to human Tregs. We identified that Tregs from cGVHD patients who responded to low-dose IL-2 demonstrated reduced expression of the gene encoding ACC1 (*Acaca*), which corresponded with increased expression of the genes encoding CD25, ICOS, and BATF, similar to the signature we identified in murine Tregs. As in murine experiments, ACC1 inhibition boosted the suppressive potency of human Tregs in vitro and in a xenogeneic GVHD model. To our knowledge, this is the first study that demonstrates a central role for mitochondrial fusion in modulating and enhancing the Treg suppressive phenotype.

Previous studies in hepatocytes, cardiomyocytes, and immortal cell lines indicate that ACC1 supplies malonyl-CoA for de novo lipogenesis (DNL), while ACC2-mediated malonyl-CoA production inhibits CPT1a and FAO (27, 68). However, our data are in line with previous work in T cells (29, 31), and suggest that ACC2 does not play a role in regulating Treg suppressive or metabolic activity (Supplemental Figure 2, E–G), and that ACC1 may be responsible for suppressing CPT1a and FAO (Figure 6, C–F, H, and I). This is further supported by preliminary data with C75, a pharmacologic inhibitor of fatty acid synthetase, the DNL rate-limiting enzyme. Unlike ACC1 inhibition, C75 pretreatment did not enhance Treg in vitro suppressive function or OXPHOS capacity (Supplemental Figure 9, B–D), indicating that the effect of ACC1 inhibition is not driven by reduced DNL. Rather, we posit that the major role for ACC1 in Tregs is malonyl-CoA–mediated suppression of CPT1a and FAO, and that ACC1 inhibition releases this inhibitory effect, thereby enhancing mitochondrial metabolism. While this may be a new concept in T cells, these findings are consistent with one of the original characterizations of malonyl-CoA activity in suppressing FAO (28).

Given the association between ACC1, FAO, and Treg suppressive function (24, 34, 69–71), we posit that ACC1 inhibition drives a network of downstream mitochondrial and gene expression changes that instruct Tregs to acquire a highly suppressive phenotype. This concept is similar to recent work showing that FAO is important for generating a pool of highly functional “effector” Tregs (72), supporting the idea that tipping Treg metabolism toward FAO maximizes the utilization of metabolic pathways favorable for supporting Treg suppressor function. The downstream results of this shift toward FAO are multifaceted. Flow cytometry analysis and unbiased RNA sequencing results show that ACC1 inhibition directs upregulation of the transcription factors T-bet and BATF, which are crucial for optimal effector function, and increased cell surface expression of TIGIT, LAG3, IL-10, CD25, and ICOS, which may work in concert to drive the functional augmentations we observed. Indeed, combinatorial, but not individual, blockage of TIGIT, LAG3, and IL-10 diminished the effect of ACC1 inhibition on Treg in vitro suppression, suggesting a cooperative mechanism of enhanced suppression between contact-dependent and contact-independent Treg suppressive molecules.

Treg coexpression of T-bet and TIGIT, observed after ACC1 inhibition, may also be particularly pertinent, as coexpression is associated with a highly suppressive Treg phenotype, and recent work demonstrated the importance of OXPHOS driving Treg TIGIT expression (72–77). Similarly, BATF has recently been identified as a key transcription factor in regulating Treg function within tissues and tumors (35, 40), along with a subset of “effector” Tregs with high TIGIT expression (78); notably, a direct interplay between BATF and T-bet is well described in the CD8^+^ T cell literature (79), and may be an additional cooperative interaction after ACC1 inhibition in Tregs. Furthermore, recent work has shown that enhanced OXPHOS can increase BATF expression in naive T cells (80), and in iTregs, ACC1 inhibition can induce epigenetic and gene expression changes (31), further suggesting that augmenting mitochondrial function may directly modulate Treg expression patterns. Collectively, these findings may reflect a cooperative role between Treg metabolism and gene transcriptional networks that induce a highly functional Treg phenotype.

Our data indicate that mitochondrial fusion may be a key convergence point for upstream metabolic modulations resulting in Treg functional reprogramming. Mitochondrial morphology, particularly the degree of fused and elongated mitochondria, depends on the overall cellular metabolic state, such that nutrient-rich (anabolic) states favor fission, whereas nutrient-sparse (catabolic) states favor fusion and elongation (81). As such, we hypothesize that after ACC1 inhibition, enhanced FAO and OXPHOS drive a catabolic state that favors mitochondrial fusion. In Tcons, M1/MDIVI1–induced mitochondrial fusion drives memory cell formation and, as in Tregs, reinforces an FAO-driven OXPHOS metabolic phenotype that works in concert to enhance in vivo function (54). These findings in memory T cells, which share a similar metabolic profile to Tregs, parallel our results seen with ACC1 inhibition and M1/MDIVI1, and suggest that shifting of Treg metabolism to favor FAO and OXPHOS may be the driver for mitochondrial fusion. Furthermore, our finding that M1/MDIVI1 treatment and ACC1 inhibition resulted in near-identical increases in metabolic and suppressive function further supports mitochondrial fusion as a key downstream pathway that mediates Treg functional reprogramming.

Finally, our data from human Tregs provide foundational evidence that ACC1 inhibition may have direct translational potential by enhancing human Treg therapeutic efficacy. Our gene expression profiling of Tregs derived from patients with cGVHD indicates that alterations in ACC1 expression may already play a role in cGVHD treatment responses. Results in our xenogeneic GVHD model further suggest that direct ACC1 inhibition in Tregs may be an efficacious approach to enhance Treg-mediated prevention of GVHD. These studies with ND630 are particularly relevant for human translation, since ND630 is already being used in clinical trials (33). Beyond GVHD, there is new interest in, and opportunities to utilize, chimeric antigen receptor (CAR) Tregs for treatment of inflammatory disorders, including GVHD, and malignancies (82, 83). By exploiting metabolic manipulations to augment CAR Treg capacity, there may be opportunities to enhance CAR Treg therapeutic potential in the future as well.

## Methods

Additional methods are described in Supplemental Methods.

### Sex as a biological variable.

Our study examined male and female mice and human patients, and no discernible differences between sexes were identified.

### Mice.

C57BL/6 (B6), B6 CD45.1, and BALB/c mice were purchased from Charles River Laboratories. B10.BR mice, Mito-Dendra2 mice [B6;129S-Gt(ROSA)26Sor^tm1.1(CAG-COX8A/Dendra2)Dcc^/J], and NOD/Scid/IL-2γc^–/–^/IL-3/GM/SF mice were purchased from The Jackson Laboratory. ACC1-deficient mice (ACC1^fl/fl^ × FoxP3-YFP-Cre) were provided by Tim Sparwasser (Johannes Gutenberg University, Mainz, Germany). CPT1a-deficient mice (CPT1a^fl/fl^ × CD4-Cre) were generated from CPT1a^fl/fl^ and CD4-Cre strains, both from The Jackson Laboratory. ACC2-deficient mice (ACC2^fl/fl^ × FoxP3-YFP-Cre) were generated from ACC2^fl/fl^ and FoxP3-YFP-Cre strains, both from The Jackson Laboratory. All mice were housed in a specific pathogen–free facility and used between 6 and 12 weeks of age. All mice were used with the approval of the University of Minnesota IACUC.

### Mouse T cell isolation and inhibitor treatments.

Tcons were purified from spleens by negative selection using biotin anti-CD19 (1D3), anti-CD11b (M1/70), anti-CD11c (N418), anti-NK1.1 (PK136), anti-CD49b (DX5), anti-CD25 (PC61.5), and anti–TER-119 antibodies (Stemcell Technologies), followed by streptavidin RapidSpheres depletion with EasySep magnet (Stemcell Technologies). Tregs were purified from lymph nodes and spleens as previously described (41), with greater than 97% Foxp3^+^ purity after CD25 enrichment (Supplemental Figure 1D).

For inhibitor treatments, Tregs were resuspended to 1 × 10^6^ to 2 × 10^6^ cells/mL in RPMI 1640 (Thermo Fisher Scientific) supplemented with 10% FBS (Thermo Fisher Scientific), 10 mM HEPES (MilliporeSigma), 2 mM glutamine (Thermo Fisher Scientific), 1× penicillin/streptomycin (MilliporeSigma), 1× non-essential amino acids (Fisher Scientific), 50 μg/mL gentamicin sulfate (Corning), and 55 mM 2-mercaptoethanol (MilliporeSigma). After resuspension, Tregs were treated for 2 hours at 37°C/5% CO_2_ with 10 μM ND630 (MedChem Express), 30 μM TOFA (Millipore), 200 nM Soraphen A (Sun-Shine Chemical), 10 μM CD-017-0191 (Millipore), 10 μM C75 (Millipore), 10 μM IACS-010759 (Cayman Chemical), 20 μM M1 with 10 μM MDIVI1 (both from Millipore), 20 μM MFI8 (MedChem Express), 5 μM etomoxir (Selleck Chemicals), a combination of 10 μM ND630 with 10 μM IACS-010759, a combination of 10 μM ND630 with 20 μM MFI8, a combination of 10 μM ND630 with 5 μM etomoxir, or an equivalent amount of DMSO. All inhibitors were reconstituted or diluted in DMSO, aliquoted at 10× final concentration, and stored at –80°C before thawing and use.

### Murine GVHD models.

A B6→BALB/c acute GVHD (aGVHD) model was used as previously described (41, 42). Briefly, BALB/c mice were irradiated with 6.0 Gy total-body irradiation (TBI) on day –1 and then injected i.v. on day 0 with 10^7^ B6 BM cells ± 1.5 × 10^6^ B6 Tcons alone or Tcons plus 0.75 × 10^6^ B6 Tregs. Mice were monitored daily for survival. Clinical scores and weights were obtained twice weekly through 4 weeks after transplant, and then weekly thereafter. Clinical scores were obtained as previously described (84). A B6→B10.BR chronic GVHD model was used as previously described (43, 44). Briefly, B10.BR recipients were conditioned with 120 mg/kg cyclophosphamide (days –3, –2) and 7.6 Gy TBI (day –1). Recipients received 10 × 10^6^ purified BM cells ± 73.5 × 10^3^ B6 purified Tcons on day 0. Additional groups of mice were given 0.5 × 10^6^ Tregs on D28 after transplant, as previously described (45). Mice were monitored daily for survival, and weights were obtained weekly. Pulmonary function tests were obtained between days 50 and 56 after transplant (46). Briefly, mice were anesthetized and ventilated using the Flexivent system (Scireq). Pulmonary resistance, elastance, and compliance were measured using Flexivent Software v5.1.

### Xenogeneic GVHD model.

Xenogeneic GVHD was used as previously described (66, 67). Briefly, NSG-SGM3 mice were treated with 0.5 Gy TBI, then injected with PBMCs (2.5 × 10^6^) ± expanded Tregs (5 × 10^6^) pretreated with DMSO or ND630. Mice were assessed daily for survival, and clinical GVHD scores and weights were obtained 3 times weekly.

### Statistics.

Data are reported as mean values ± SEM. Pairs were compared using unpaired 2-tailed Student’s *t* tests with Bonferroni’s multiple-comparison correction, as needed. Data sets with 3 or more samples were compared using 1-way ANOVA with multiple-comparison analysis, including a Tukey’s post hoc test with multiple-comparison correction. Differences in survival were analyzed by log-rank test. Significance was defined as *P* less than 0.05. Statistical analyses were performed using Prism v9 (GraphPad).

### Study approval.

Animal studies were conducted in accordance with a protocol reviewed and approved by the IACUC of the University of Minnesota (2103-38905A). Patient samples for RNA sequencing were collected as part of IRB-approved clinical trials at the Dana-Farber Cancer Institute (NCT02318082 and NCT01366092). Prior written informed consent was obtained per the Declaration of Helsinki. Human cells utilized for in vitro studies were derived from leukapheresis products purchased from the Memorial Blood Center (St. Paul, Minnesota, USA). No approval was required for these purchased human samples.

### Data availability.

The murine RNA sequencing generated in this study is publicly available in the NCBI’s Gene Expression Omnibus (GEO) database under accession number GSE294218. The human RNA sequencing used in this study is publicly available in GEO under accession number GSE296708. All supporting data values are compiled in the Supporting Data Values file.

## Author contributions

CMH designed and performed experiments, discussed results, and wrote the paper. EGA designed and performed experiments, discussed results, and contributed to writing the paper. EBC, SYR, FAM, MCZ, JHL, MLL, CL, and GT designed and performed experiments, discussed results, and edited the paper. MLS, KDS, and JSW performed experiments and edited the paper. JR, TDS, RSO, JCR, PAC, RZ, LSK, KLH, MLD, and BRB contributed to experimental design, discussed results, and edited the paper.

## Funding support

This work is the result of NIH funding, in whole or in part, and is subject to the NIH Public Access Policy. Through acceptance of this federal funding, the NIH has been given a right to make the work publicly available in PubMed Central.

- National Heart, Lung, and Blood Institute and National Institute of Allergy and Infectious Diseases awards R37AI34495, P01HL158505, and R01HL11879 (to BRB); 2T32HL007062 (to CMH); and 1P01HL158505 (to JR).
- European Research Council awards ERC-2015-AdG_670930 and ERC-2021-SyG_951329 (to EBC and MLD), and ERC-2022-ADG_101094168 (to RZ).
- German Research Foundation awards SFB-1479_441891347 and 872/4-1 (to RZ), and SFB1292_31834696 and SFB355_490846870 (to TDS).
- The University of Minnesota Characterization Facility receives support from the National Science Foundation through the Materials Research Science and Engineering Center (DMR-2011401) and the National Nanotechnology Coordinated Infrastructure (ECCS-2025124).

## Supplementary Material

Supplemental data

Supporting data values

## Version 1 09/30/2025

In-Press Preview

## Version 2 12/01/2025

Electronic publication
