# Supplementary material for: Acetyl-CoA carboxylase 1 inhibition increases Treg metabolism and graft-versus-host disease treatment efficacy via mitochondrial fusion
Source: J Clin Invest. 2025 Sep 30;135(23):e182480. doi: 10.1172/JCI182480 (PMC12646659; doi:10.1172/JCI182480)
Supplement: Supplemental data [file jci-135-182480-s205.pdf]

Supplemental Methods

Suppression assays and CFSE staining. Murine in vitro suppression assays were conducted as described (1), including calculating the % Treg suppression using the following formula: [(100 - % Tcon proliferation in the present of Treg)/(%Tcon proliferation in the absence of Treg)]*100. In certain experiments, blocking monoclonal antibodies against TIGIT (1B4, Thermo), Lag3 (C9B7W, Thermo), IL-10 (JES5-2A5, Thermo), or IL-10R (1B1.3A, BioXcell) were added to cultures. Anti-TIGIT was used at 25µg/mL, anti-Lag3 at 60µg/mL, anti-IL-10 at 10µg/mL and anti-IL-10R at 10µg/mL; isotype controls were added at the same concentration. For human suppression assays, PBMCs (Memorial Blood Center, St. Paul, MN) were purified, labeled with CFSE as above, and mixed with Treg in the presence of anti-CD3 coated beads (Dynal). PBMCs were mixed with control or treated Treg at Treg:Tcon ratios of 0:1, 1:9, 1:27 and 1:81. At least 3 biological replicates per assay. CFSE dilution was analyzed after 96 hours by flow cytometry.

For in vivo assessment of CFSE dilution, freshly purified $CD4^+CD25^-$ and $CD8^+CD25^-$ B6 Tcon were labeled with CFSE for 20 minutes at 37°C prior to infusion. A total of 10^7 Tcon were infused into TBI conditioned BALB/c recipients. Some mice also received $5x10^6$ WT or ACC1KO Treg. Recipient spleens were harvested 72 hours after cell infusions and analyzed by flow cytometry for Tcon CFSE dilution. A portion of splenocytes were also stimulated for cytokine analysis by flow cytometry.

Mouse Treg Stimulation. For suppressive molecule analysis, electron microscopy, metabolic flow and Seahorse assays, freshly purified Treg were treated with inhibitors as above, then resuspended in complete-RPMI at $1x10^6$ cells/mL with 1000IU/mL recombinant human IL-2 (rH-IL-2,Novartis). $1x10^6$ cells/well were stimulated for ~18hr in the at 37ºC/5%CO_2 in a 24-

well tissue-culture treated plate coated with 10µg/mL each of anti-CD3 (145-2C11) and anti-CD28 (37.51) mAb. For phosphoflow, Treg were stimulated for 60min in a tissue-culture treated 96-well round bottom plate. For cytokine analysis, cells were incubated for 5 hours at 37°C with 1x eBioscience Cell Stimulation Cocktail prior to staining.

Human Treg culture. Human Treg (CD4+CD25hiCD127loCD45RA+) were sorted from leukapheresis products (Memorial Blood Center, St. Paul, MN). Sorted cells were incubated with irradiated K562 cells line (KT86/64) as previously described(2). Treg were cultured in XVivo-15 medium (Lonza) containing 10% human serum (Valley Biomedical), 1x Pen/Strep and GlutaMAX (Thermo), N-acetyl cysteine (USP), and rH-IL-2 (300 IU/mL). After 14 days, cells were treated with either DMSO or ND630 and then utilized in assays.

Seahorse metabolic flux assays. OCR and ECAR were measured using the XF-96 Extracellular Flux Analyzer (Seahorse Bioscience) in XF media (DMEM/2mM L-glutamine/1mM sodium pyruvate/2.5mM glucose for OCR; DMEM/2mM L-glutamine for ECAR), per manufacturer protocols. OCR was measured under basal conditions and in response to 1.5µM oligomycin, 1µM fluorocarbonyl cyanide phenylhydrazone or 2.5µM BAM15, and 0.5µM rotenone/0.5µM antimycin-A. ECAR was measured under basal conditions and in response to 10mM Glucose, 1µM oligomycin, 50mM 2-deoxyglucose. $0.3x10^6$ Treg per well were plated in quadruplicate in Cell-Tak (Corning) coated 96 well Seahorse microplates. Treg were rested at ambient CO_2/37ºC for 1hr prior to the assay. In some assays, Treg were resuspended in XF media containing 300nM of BSA complex or 300nM of BSA-palmitate (Cayman Chemical) prior to the resting period, and kept in this media for the assay.

siRNA treatment. Treg were transfected by electroporation as described (1, 3). Briefly, Treg were transfected with either 20μM SmartPool mouse MFN1 siRNA (Horizon Discovery), or SmartPool non-targeting control siRNA (Horizon Discovery). Electroporation was performed using the P3 primary cell 4D-nucleofector kit (Lonza) and a Nucleofector-4D machine (Lonza) on setting DN-100. After electroporation, Treg were transferred to 1 12-well tissue-culture treated plate containing 2mL of warmed complete-RPMI with 1000IU/mL of rH-IL-2. Treg were rested at 37ºC/5%CO_2 for 4hr. After resting, Treg were transferred to 12-well plates pre-coated with 10μg/mL each of anti-CD3/CD28 mAb. Assessment of protein knockdown and function were completed 72hr after electroporation.

Flow cytometry. Single-cell suspensions were recorded on a BD LSRFortessa flow cytometer. FCS3.0 files were analyzed using FlowJoV10.9 (Treestar Inc.). Anti-mouse antibodies were primarily obtained from Thermo or Biolegend, including: anti-CD4 (RM4-5/GK1.5), CD8 (53-6.7), CD19 (1D3), CD25 (PC61.5), CD138 (281-2), CD45.1 (A20), CD45R/B220 (RA3-6B2), Thy1.1 (HIS51), CTLA-4 (UC10-4B9), ICOS (7E.17G9), Lag3 (C9B7W), TIGIT (GIGD7), PD1 (29F.1A12), CXCR5 (SPRCL5), GL7 (GL7), FAS (Jo2), IL-10 (JES5-16E3), Foxp3 (FJK-16s), Tbet (4B10), IFN-γ (XMG1.2), and TNFα (MP6-XT22). Anti-CPT1A (8F6AE9) and anti-Glut1 (EPR3915) were both from Abcam. Polyclonal antibodies against phospho-Drp1 (Ser676) were obtained from Cell Signaling Technologies, and polyclonal antibodies against FABP5 were obtained from Biorbyt. Anti-human antibodies included the following from BD: CD4 (RPA-T4), CD8 (RPA-T8), CD25 (M-A251), CD45RA (HI100). Anti-human Foxp3 (249D) was purchased from BioLegend. Cells were stained with Fixable Viability Dye ef780 (Thermo) for all

experiments. For some experiments, cells were surface stained, then incubated with MitoTracker Deep Red, Mitotracker Green, CellRox, MitoSox, 2-NBDG, BoDipy493/503, or BoDipy$_{C16}$FL-C (all from Thermo), per manufacturer's protocols. Fixation and intracellular/intranuclear staining were done using either the eBioscience Foxp3 staining kit, or the eBioscience IC fixation kit. For phosphoflow, cells were stained using a modified version of the eBioscience Protocol B.

Tissue preparation and histochemistry. At time of euthanasia, mouse lungs were intratracheally inflated with 75% Tissue-Tek optimal cutting temperature compound (OCT), embedded in OCT, snap frozen in liquid nitrogen, and stored at -80ºC. Frozen blocks were cut in 5µm sections, mounted on microscope slides, fixed overnight, and stained using Masson trichrome (HT15-1KT; Millipore). Trichrome images were analyzed using EBImage (4).

Electron microscopy. Overnight activated Treg pre-treated with DMSO or ND630 were pelleted, and fixed with 2.5% glutaraldehyde in 0.1M sodium cacodylate. After washing with 0.1M sodium cacodylate, Treg were post-fixed with 1% osmium tetroxide for 1hr, then dehydrated in a graded series of ethanol. The samples were embedded in Epon 812 resin. 65 nm sections were stained with uranylacetate and lead citrate, then examined by JEOL 1200EX electron microscopy.

3D structured illumination and expansion microscopy. Activated Treg were bound on planar supported lipid bilayers then fixed with 3.7% paraformaldehyde (Thermo) in PHEM buffer for 10min at 37ºC, washed with PBS, and permeabilized with 0.1% Triton X-100 (Millipore) for 30min at RT. Image-IT signal enhancer (Thermo) was used prior to staining overnight at 4ºC

with 1:100 anti-vimentin Alexa Fluor 488. After extensive washing, channels are flooded with immersion-oil to optimize refractive index for 3D-SIM. Images were acquired at 21°C on a DeltaVision OMX V3 Blaze microscope (GE Healthcare, UK) with a 60x/1.42 oil UPlanSApo objective (Olympus), 405nm, 488nm, 593nm diode lasers and sCMOS cameras (PCO). 3D-SIM image stacks were acquired with 5 phases/3 angles per image plane and 0.125µm z-distance between sections. The raw data was computationally reconstructed with SoftWoRx6.0 (Applied Precision) using Wiener filter settings 0.002 and channel specifically measured optical transfer functions to generate a super-resolution 3D image stack with a lateral (x-y) resolution between 100-130nm (wavelength-dependent) and an axial (z) resolution of ~300nm (5). Images from the different color channels were registered with alignment parameter obtained from calibration measurements with nuclear pore complexes stained with all three colors provided by Micron. Images were processed using Fiji (Image J) and Imaris (Bitplane).

Mitochondrial imaging measurements. The mitochondrial sphericity, circularity (for electron microscopy images), area and integrated density were obtained using Bitplane Imaris software (v8.1.2, Bitplane AG, Switzerland, http://bitplane.com). Mitochondria were segmented using the spot extraction tool followed by generated 3D surface rendering of the mitochondria based on the intensity of their fluorescent signal. The spot extraction segmentation allowed us to identify any possible automatic segmentation issues due to overcrowding, but the high SIM resolution provides sufficient pixels in dense locations to manually identify and segment fused (continuous low “donut shaped” fluorescent signal along interaction side) or side-by-side mitochondria that were not fused together (peak fluorescent intensity where both mitochondrial membranes are near each other). Every 3D render was assigned a unique ID number and morphological features

of the surfaces were extracted. Comparisons relating to mitochondrial morphology were evaluated using both individual mitochondria and converged datapoints per cell. Circularity was measured using the following formula, with area and perimeter derived from Bitplane evaluation: $4\pi*(\text{Area}/\text{Perimiter}^2)$. Sphericity was measured using the formula below, where V_p = volume of the mitochondria and A_p = surface area of the mitochondria, both of which were derived from Bitplane evaluation:

$$\Psi = \frac{\pi^{1/3}(6V_p)^{2/3}}{A_P}$$

Murine RNA Sequencing Preparation and Analysis. Overnight activated Treg pre-treated with DMSO or ND630 were separated into samples containing at least 100,000 cells, pelleted, and resuspended in Qiagen buffer RLT with 1% 2-mercaptoethanol (Millipore). Samples were snap frozen on dry ice, and stored at -80ºC until processing at University of Minnesota Genomics Center. RNA was collected and isolates quantified by fluorometric RiboGreen assay, integrity determined using capillary electrophoresis and samples converted to sequencing libraries using Takara Bio's SMARTer Stranded Total RNA-Seq–Pico Mammalian Kit v2.

Indexed libraries were normalized, pooled, and loaded onto a NovaSeq paired end flow-cell for clustering and sequencing using Illumina's fastqv2.20. FASTQ reads were entered into the Collection of Hierarchical UMII/RIS Pipelines (6) for quality control (7), trimming, confirming viability, aligning reads (8) using HISAT2 (9), filtering alignments using SAMtools (10) and generating output matrices using Subread FeatureCounts (11). Genome alignments were run against the mm10 reference genome (GRCm38). Resulting counts were analyzed for

differential gene expression using DEseq2 (12). Secondary analyses such as GSEA and GRNA were run with Piano (13) and DIANE (14) respectively.

Human RNA sequencing Sample Preparation and Analysis. Cryopreserved peripheral blood mononuclear cell (PBMC) samples were selected from patients with refractory chronic GVHD enrolled on low-dose IL-2 treatment trials at the Dana-Farber Cancer Institute in Boston, MA (NCT02318082 and NCT01366092). PBMCs from 6 clinical responders and 5 non-responders at baseline (pre-treatment with low-dose IL-2) and after 4 weeks of low-dose IL-2 therapy were sorted into CD4Treg and CD4Tcon cells. CD4+ T cells were isolated by negative selection using a MACS® Separator and were subsequently labeled with the following antibodies: CD4-FITC (clone: OKT4), CD25-PE-Cy7 (clone: MA251), and CD127-APC-eF780 (clone: eBioRDR5). The labeled CD4+ T cells were then sorted into CD4+ CD25hi CD127lo (CD4Treg) and CD4+ CD25lo (CD4Tcon) populations using a BD FACSAria Cell Sorter. Total RNA was extracted from the sorted cells using TRIzol Reagent (Thermo Fisher Scientific) following the manufacturer's protocol.

cDNA was synthesized using Takara SmartSeq v4 reagents. Full length cDNA was fragmented to a mean size of 200bp with a Covaris R230 ultrasonicator and Illumina sequencing libraries were prepared from 2ng of sheared cDNA using IDT DNAseq library prep reagents on a Beckman Coulter Biomek i7 according to manufacturer’s protocol. The finished dsDNA libraries were quantified by Qubit fluorometer and Agilent TapeStation 2200. Uniquely dual indexed libraries were pooled in equimolar ratios and evaluated for cluster efficiency and pool balance with shallow sequencing on an Illumina MiSeq. Final sequencing was performed on an Illumina

NovaSeq X Plus with single-end 150bp reads at the Dana-Farber Cancer Institute Molecular Biology Core Facilities.

Resulting reads were trimmed, assessed for viability, and aligned using STAR 2.7.9a. Genome alignments were made against the hg38 (GRCh38) reference genome. Resulting counts were analyzed for differential gene expression using DEseq2. Results were analyzed to detect enrichment of differentially expressed genes from murine analysis within human dataset via comparison of relative fold change. Gene set enrichment analysis (GSEA) was run using Piano. Analysis was run blind against a filtered selection of gene sets from the Molecular Signature Database. The database was filtered for gene sets containing the following relevant terms for analysis so as to prevent off target enrichment: FattyAcid, Oxidative, Mitochondria, Autoimmune, GVHD, Lymphocyte, Treg, Allo, Graft, Lupus, Leukemia, Lymphoma. Results were compared against comparable analyses performed on the murine dataset, and the overlapping differentially expressed genes, and enriched gene sets were determined.

References

1. McDonald-Hyman C, Muller JT, Loschi M, Thangavelu G, Saha A, Kumari S, et al. The vimentin intermediate filament network restrains regulatory T cell suppression of graft-versus-host disease. *J Clin Invest.* 2018;128(10):4604-21.
2. Hippen KL, Merkel SC, Schirm DK, Sieben CM, Sumstad D, Kadidlo DM, et al. Massive ex vivo expansion of human natural regulatory T cells (T(regs)) with minimal loss of in vivo functional activity. *Sci Transl Med.* 2011;3(83):83ra41.
3. Zanin-Zhorov A, Ding Y, Kumari S, Attur M, Hippen KL, Brown M, et al. Protein Kinase C-Theta Mediates Negative Feedback on Regulatory T Cell Function. *Science.* 2010;328(5976):372-6.
4. Pau G, Fuchs F, Sklyar O, Boutros M, and Huber W. EBImage—an R package for image processing with applications to cellular phenotypes. *Bioinformatics.* 2010;26(7):979-81.
5. Schermelleh L, Carlton PM, Haase S, Shao L, Winoto L, Kner P, et al. Subdiffraction multicolor imaging of the nuclear periphery with 3D structured illumination microscopy. *Science.* 2008;320(5881):1332-6.
6. Baller J, Kono T, Herman A, and Zhang Y. *ACM International Conference Proceeding Series*. 2019.
7. Andrews S. Babraham Bioinformatics - FastQC A Quality Control tool for High Throughput Sequence Data. https://www.bioinformatics.babraham.ac.uk/projects/fastqc/.

8. Bolger AM, Lohse M, and Usadel B. Trimmomatic: a flexible trimmer for Illumina sequence data. *Bioinformatics.* 2014;30(15):2114-20.
9. Kim D, Paggi JM, Park C, Bennett C, and Salzberg SL. Graph-based genome alignment and genotyping with HISAT2 and HISAT-genotype. *Nature Biotechnology.* 2019;37(8):907-15.
10. Danecek P, Bonfield JK, Liddle J, Marshall J, Ohan V, Pollard MO, et al. Twelve years of SAMtools and BCFtools. *GigaScience.* 2021;10(2).
11. Liao Y, Smyth GK, and Shi W. featureCounts: an efficient general purpose program for assigning sequence reads to genomic features. *Bioinformatics.* 2014;30(7):923-30.
12. Love MI, Huber W, and Anders S. Moderated estimation of fold change and dispersion for RNA-seq data with DESeq2. *Genome Biology.* 2014;15(12):550.
13. Väremo L, Nielsen J, and Nookaew I. Enriching the gene set analysis of genome-wide data by incorporating directionality of gene expression and combining statistical hypotheses and methods. *Nucleic Acids Res.* 2013;41(8):4378-91.
14. Cassan O, Lèbre S, and Martin A. Inferring and analyzing gene regulatory networks from multi-factorial expression data: a complete and interactive suite. *BMC Genomics.* 2021;22(1):387.

Supplemental Data

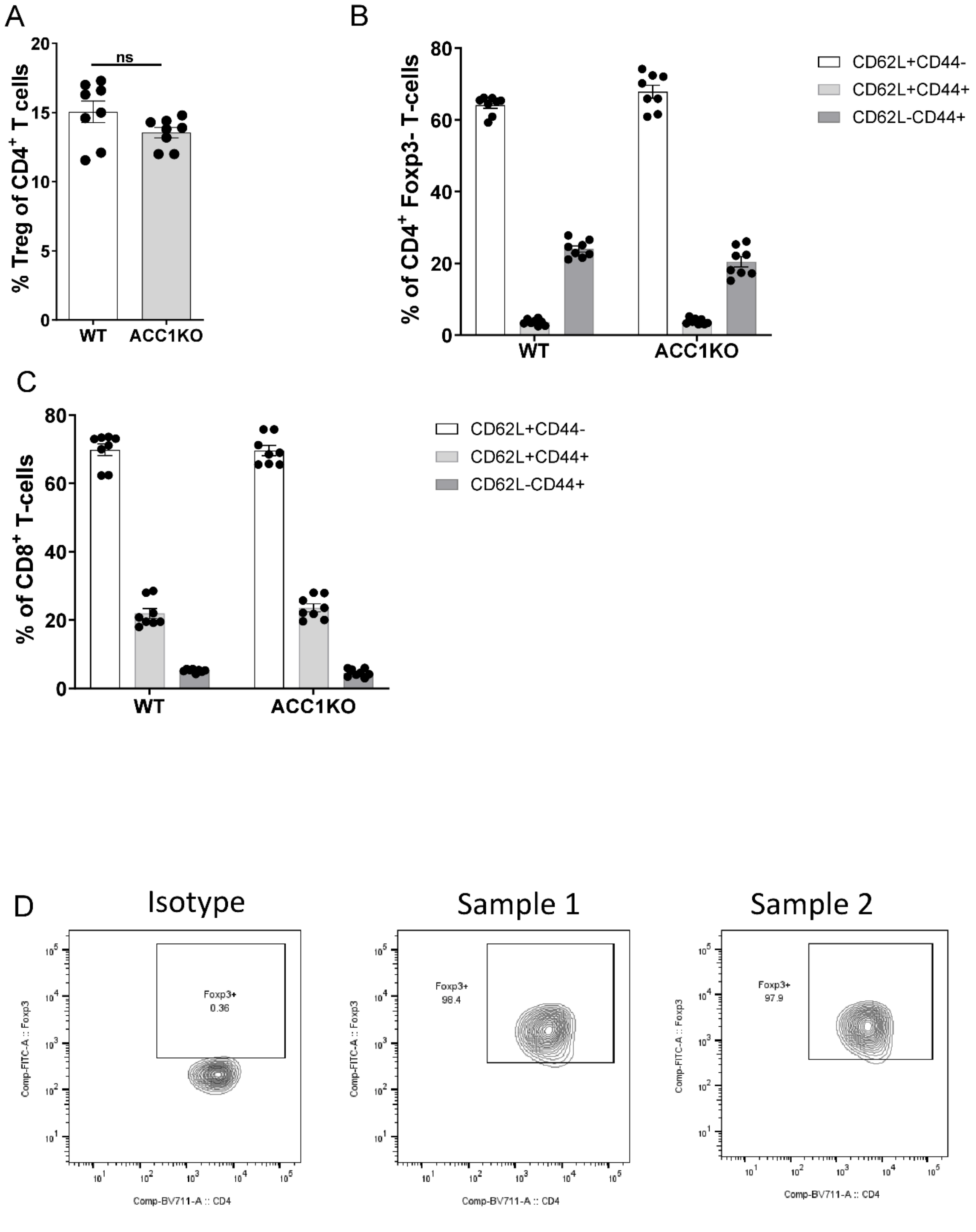

Supplemental Figure 1

Supplemental Figure 1. Characterization of T-cell populations in ACC1fl/fl x Foxp3-YFP-Cre mice. (A-C) Splenocytes from either WT B6 mice or ACC1KO B6 mice were isolated, made into a single cell suspension and stained for flow cytometry. **A**) Comparison of the % of CD25+Foxp3+ within the CD4+ T-cell population between WT and ACC1KO mice. (**B-C**) Evaluation of naïve (CD62L+CD44-), central memory-like (CD62L+CD44+) and effector memory-like (CD62L-CD44+) in B) CD4+Foxp3- T-cells and C) CD8+ T-cells. Data show one experiment representative of two independent experiments with n=8mice/group. **D**) Representative flow cytometry plots comparing the % Foxp3+ of CD4+CD25+ cells after Treg purification by CD25 enrichment. Isotype (left) was used for the gating shown. Samples 1 and 2 represent two different pools of Treg purified in an identical manner, but isolated separately. $^{*}p<0.05$; $^{**}p<0.01$; $^{***}p<0.001$; $^{****}p<0.0001$ by unpaired t-test. Error bars=SEM.

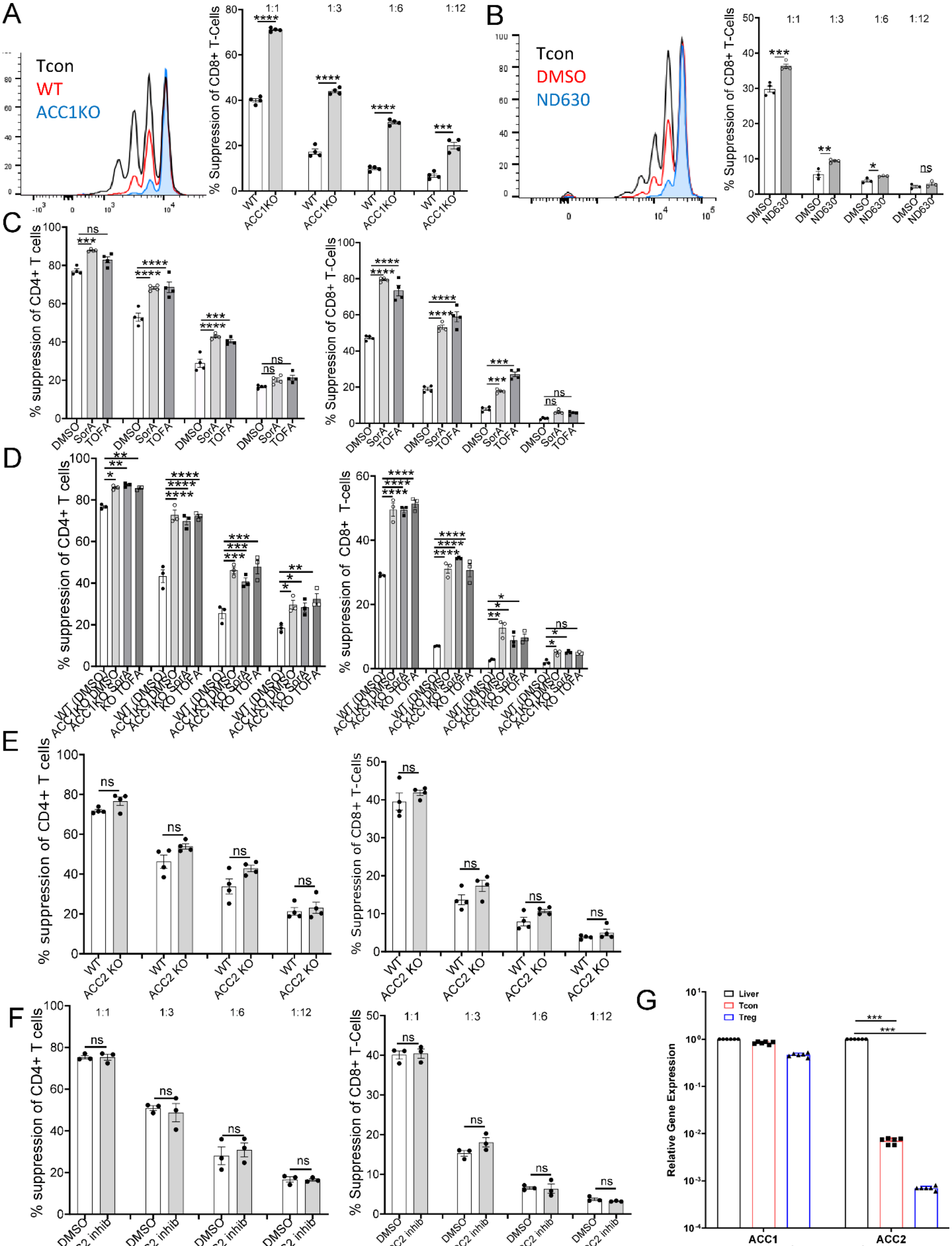

Supplemental Figure 2

Supplemental Figure 2. ACC1 but not ACC2 inhibition augments Treg *in vitro* function. (**A-B**) Suppression of CD8+ Tcon proliferation in classical *in vitro* Treg suppression assays by **A)** wild type (WT) vs. ACC1 knockout (ACC1KO) Treg, and **B)** DMSO vs. ND630 pre-treated Treg. CFSE dilution plots show Treg:Tcon ratio of 1:3. Graphs show 1:1–1:12 Treg:Tcon ratios. **(C-D)** Suppression of CD4+ (left) and CD8+ (right) Tcon proliferation in classical *in vitro* Treg suppression assays by **C)** DMSO, Soraphen A (SorA) vs TOFA pre-treated WT Treg, and **D)** DMSO, Soraphen A (SorA) vs TOFA pre-treated ACC1KO Treg. **(E-F)** Suppression of CD4+ (left) and CD8+ (right) Tcon proliferation in classical *in vitro* Treg suppression assays by **E)** wild type (WT) vs. ACC2 knockout (ACC2KO) Treg, and **F)** DMSO vs. CD-017-0191 (ACC2 inhibitor) pre-treated Treg. 1:1–1:12 Treg:Tcon ratios denoted. G**)** Expression of ACC1 and ACC2 as measured by qPCR from Tcon and Treg isolated from WT mice, as well as liver tissue isolated from the same animals. Expression is graphed relative to liver tissue expression of ACC1 and ACC2. Data show one experiment representative of 2 (qPCR analysis) or 3 independent experiments, with n = 6 technical replicates for qPCR assays, and n=4 technical replicates/group for suppression assays. *$p<0.05$; **$p<0.01$; ***$p<0.001$; ****$p<0.0001$ by unpaired *t*-test or one-way ANOVA. Error bars=SEM.

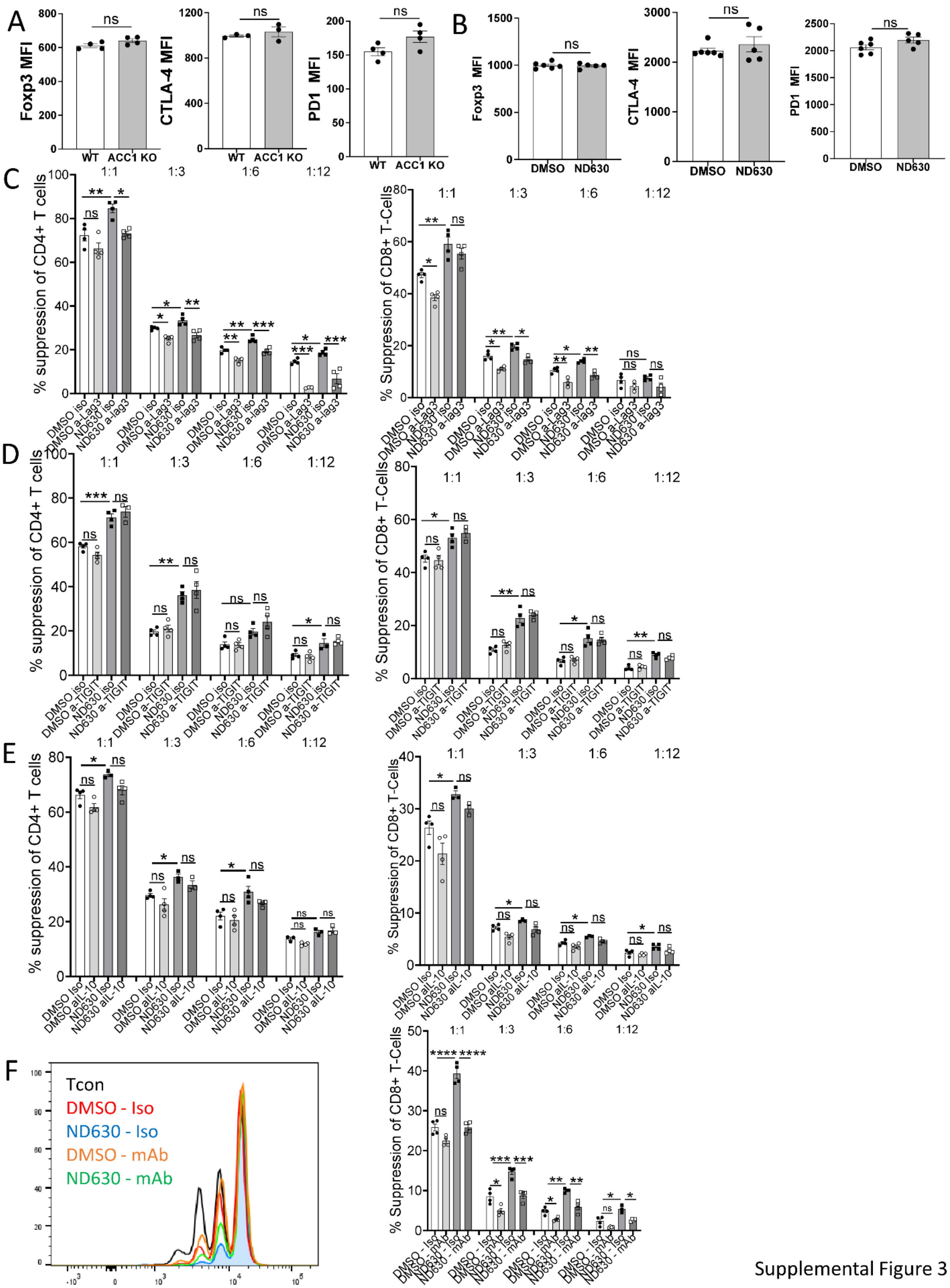

Supplemental Figure 3

Supplemental Figure 3. ACC1 inhibition does not modulate or require canonical Treg suppressive molecule expression for enhanced suppressive function. Flow cytometry evaluation of expression of Foxp3, CTLA-4 and PD1 on **A)** WT vs. ACC1KO Treg, and **B)** DMSO vs. ND630 pre-treated Treg after overnight activation with plate bound anti-CD3/28 monoclonal antibodies and IL-2, as evaluated by median fluorescent intensity (MFI). **(C-E)** Suppression of CD4+ (left) and CD8+ (right) Tcon proliferation in classical *in vitro* Treg suppression assays by DMSO or ND630 pre-treated Treg, with cultures including either **C)** isotype or anti-Lag3 blocking antibodies, **D)** isotype or anti-TIGIT blocking antibodies, **E)** isotype or anti-IL-10 combined with anti-IL-10 receptor blocking antibodies, and **F)** isotype or a combination of anti-Lag3, TIGIT, IL-10 and IL-10R blocking antibodies; CFSE dilution plot shows Treg:Tcon ratio of 1:3. 1:1–1:12 Treg:Tcon ratios denoted; n = 4/group. Data show one experiment representative of or 3 independent experiments. *p<0.05; **p<0.01; ***p<0.001; ****p<0.0001 by unpaired *t*-test or one-way ANOVA. Error bars=SEM.

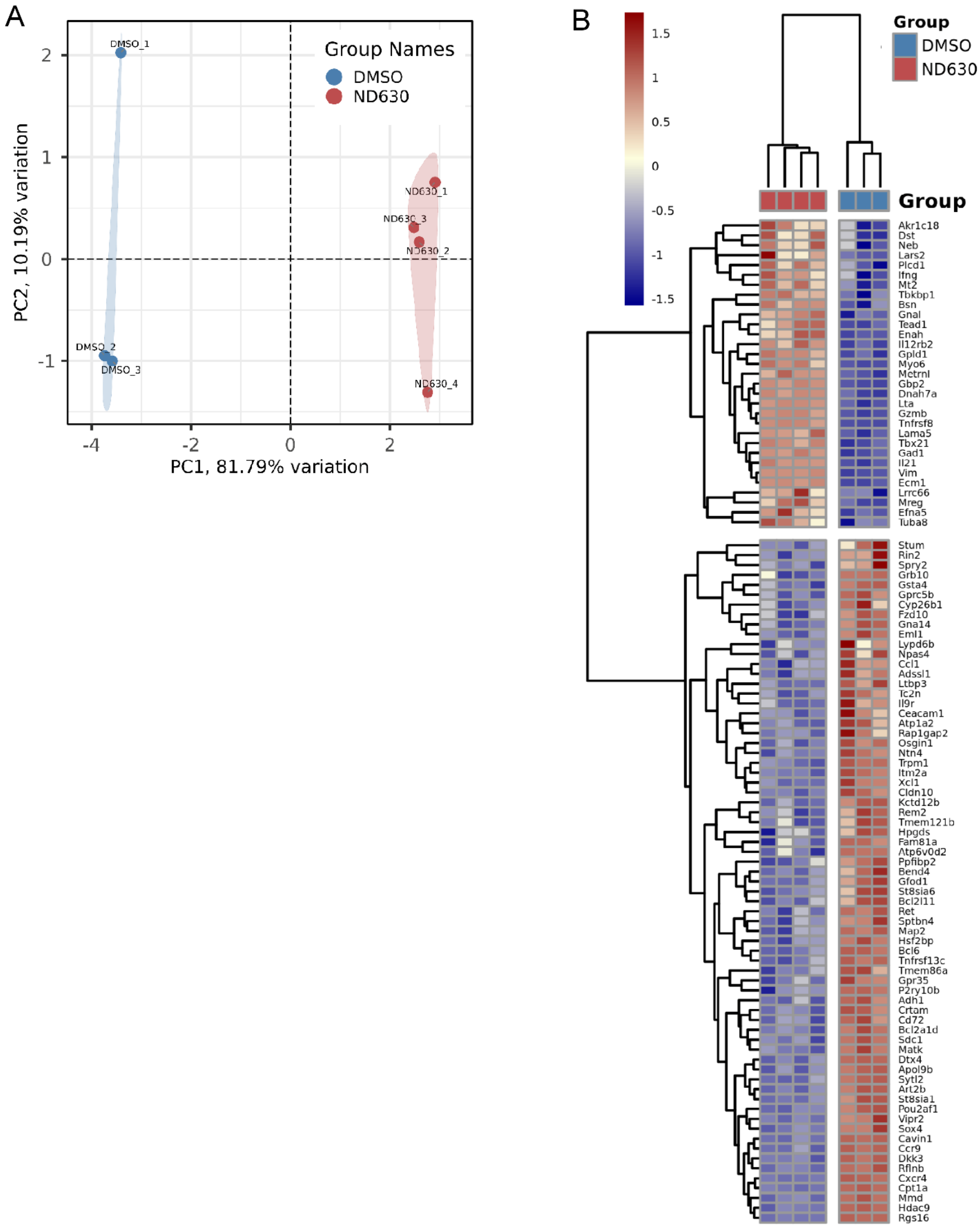

Supplemental Figure 4

Supplemental Figure 4. ACC1 inhibition alters Treg expression patterns. Bulk RNA sequencing results of Treg pre-treated with either DMSO or ND630, and then activated overnight with plate bound anti-CD3/28 monoclonal antibodies and IL-2. **A)** Principle component analysis noting independent clustering of DMSO and ND630 samples, with 81.79% of variance in gene expression between groups attributable to ND630 treatment. **B)** Heat map showing the 100 most differentially expressed genes between DMSO (blue, top clustering) and ND630 (red, top clustering). **C)** GSEA bubble plot showing pathway enrichment comparisons between the data set analyzed in **A-B** and bulk RNA sequencing from Treg isolated from patients with chronic GVHD treated low-dose IL-2. Three individual samples were collected and analyzed in the DMSO group, while 4 were collected and analyzed in the ND630 group; a total of *** patient samples were used for analysis in the human data. Differentially expressed genes defined as having an adjusted value of $P < 0.05$, and a $\log_2$ fold change greater than 0.15.

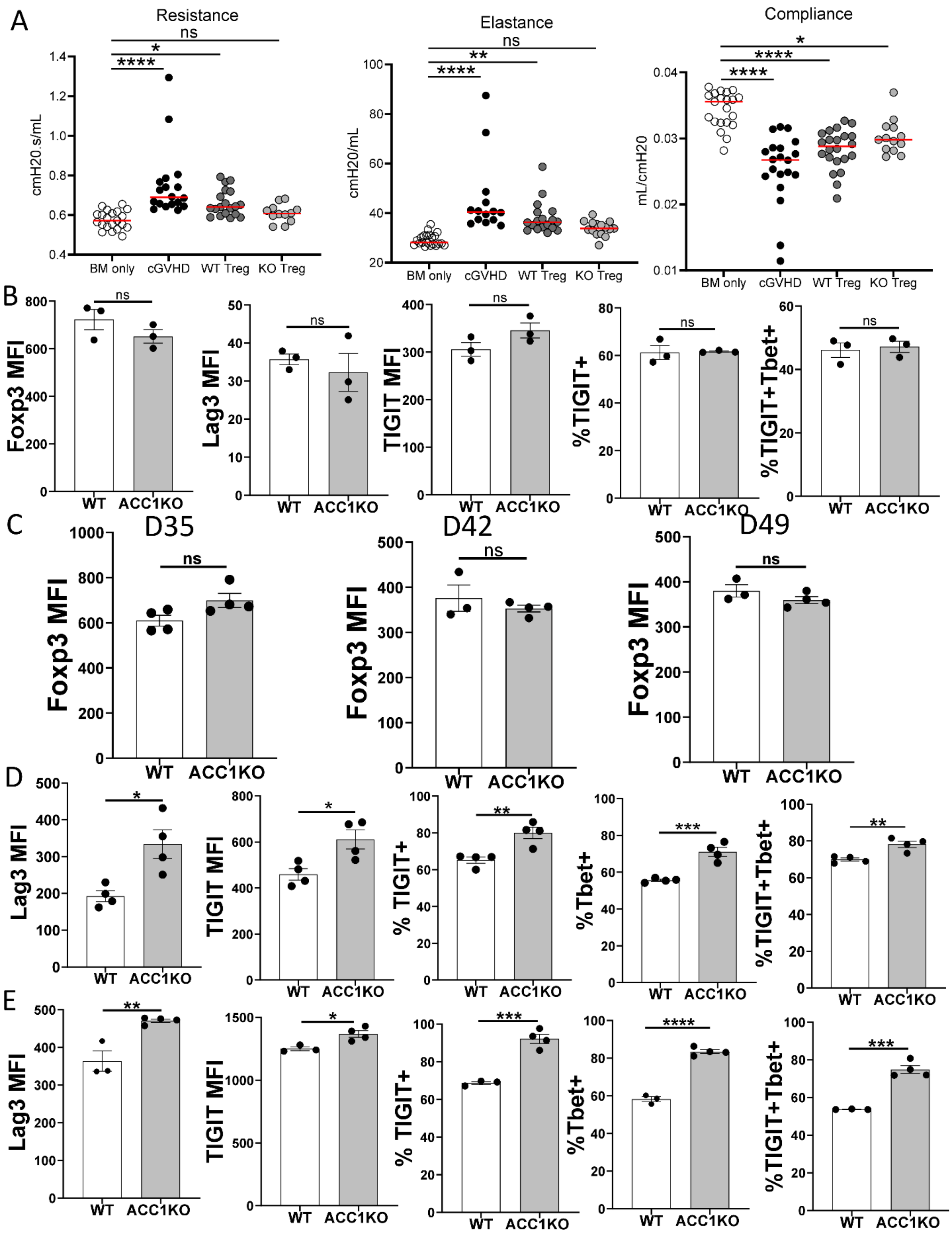

Supplemental Figure 5

Supplemental Figure 5. ACC1 inhibition alters Treg phenotype *in vivo*

Chronic GVHD transplant using cyclophosphamide/TBI conditioned B10.BR recipients receiving $10x10^6$ BM cells (BM)±$73.5x10^3$ B6 T-cells (D0). Two groups of mice given BM+T-cells, were subsequently given $0.5x10^6$ of either WT Treg or ACC1KO (KO) Treg on D28 post-transplant. **A)** Pulmonary function tests obtained on D49 post-transplant showing Airway Resistance, Lung Elastance and Total Lung Compliance of mice given either BM alone (BM only), BM+T-cells (cGVHD), or BM+T-cells with WT Treg or ACC1KO Treg. Data are pooled from 3 transplants, with n=22 BM only; n=21 cGVHD; n=20 WT Treg and n=15 ACC1KO Treg. **(B)** Flow cytometry of freshly purified, non-activated WT and ACC1KO Treg prior to infusion on D28. Expression of Foxp3, LAG3 and TIGIT, and frequency of TIGIT+, and TIGIT+Tbet+ double positive Treg within the donor CD4+CD25+Foxp3+ population are shown. Data show sub-sampling of pooled WT and ACC1KO Treg, with n=3/group. **C**) Flow cytometry showing Foxp3 MFI of donor WT and ACC1KO Treg identified in the spleen of recipient mice on D35, 42 and 49 after transplant. **D-E**) Flow cytometry of WT and ACC1KO Treg on **D**) D35 or **E**) D49 after transplant showing expression of LAG3 and TIGIT, and frequency of TIGIT+, Tbet+ and TIGIT+Tbet+ double positive Treg within the donor CD4+CD25+Foxp3+ population. Data show one experiment representative of two independent experiments, with n=3 or n=4/group. *$p<0.05$; **$p<0.01$; ***$p<0.001$; ****$p<0.0001$ by unpaired *t*-test or one-way ANOVA. Error bars=SEM. Median fluorescent intensity=MFI.

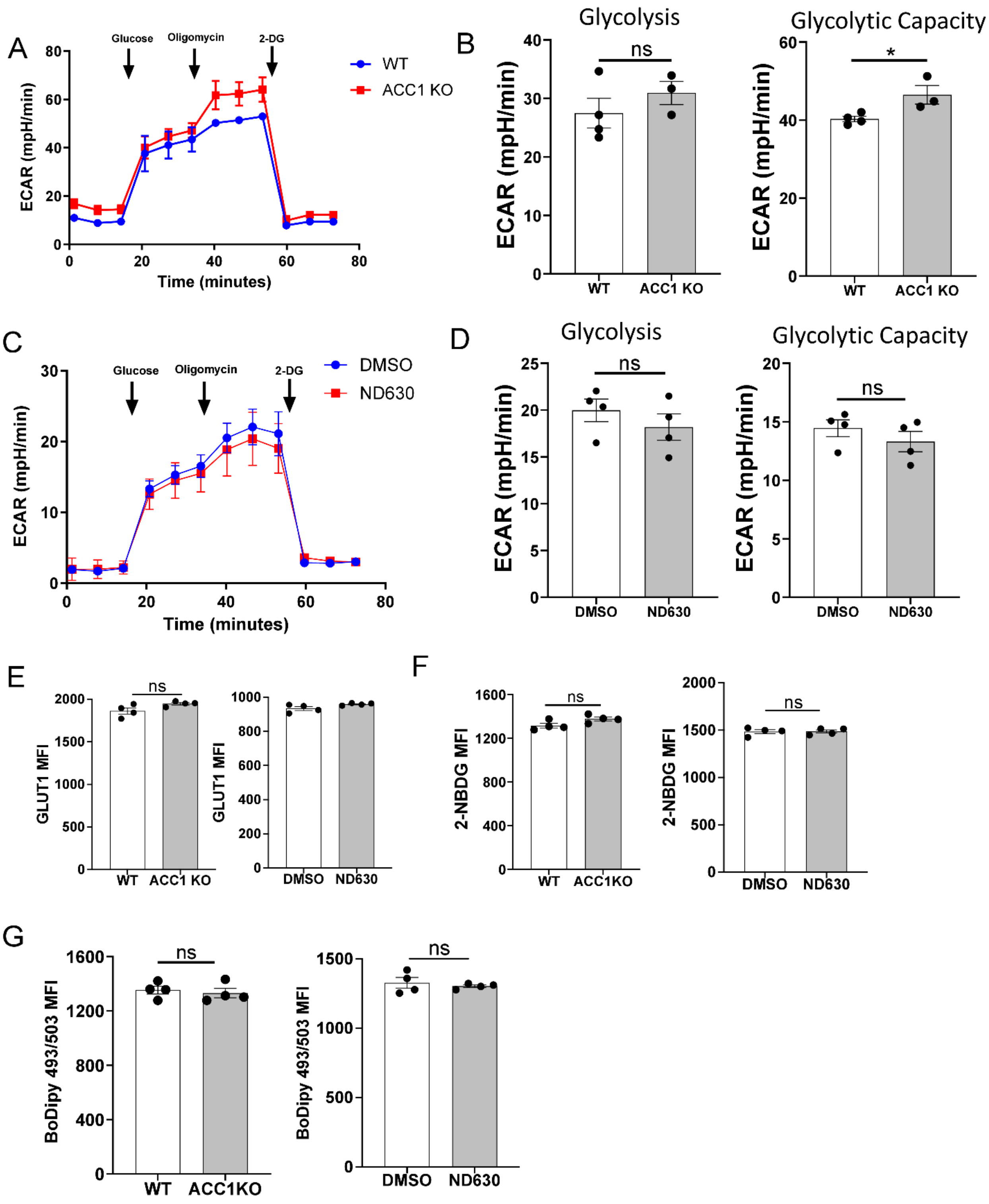

Supplemental Figure 6

Supplemental Figure 6. ACC1 inhibition does not alter glycolytic capacity or glucose uptake of Treg. (A-D) Extracellular acidification rate (ECAR) analysis in standard Seahorse glycolysis stress test assays, comparing ECAR of **(A-B)** WT and ACC1KO Treg, or **(C-D)** DMSO and ND630 pre-treated Treg following overnight activation with plate bound anti-CD3/28 monoclonal antibodies and IL-2. **A)** ECAR curves of WT vs. ACC1KO Treg in response to glucose, oligomycin and 2-deoxyglucose (2-DG), with **B)** quantification of glycolysis and glycolytic capacity for each group; n = 4/group. **C)** ECAR curves of DMSO vs. ND630 pre-treated Treg in response to glucose, oligomycin and 2-DG, with **D)** quantification of glycolysis and glycolytic capacity for each group; n = 4/group. **E)** Flow cytometry evaluation of Glut1 expression on WT vs. ACC1KO Treg (left), or DMSO vs. ND630 pre-treated Treg (right) after overnight activation with plate bound anti-CD3/28 monoclonal antibodies and IL-2. **F)** Flow cytometry evaluation of fluorescent glucose analog 2-NBDG uptake by WT vs ACC1KO Treg (left), or DMSO vs. ND630 pre-treated Treg (right) (n= 4/group) after overnight activation with plate bound anti-CD3/28 monoclonal antibodies and IL-2. G) Flow cytometry evaluation of endogenous lipid droplets using BoDipy 493/503 comparing WT vs ACC1KO Treg (left), or DMSO vs. ND630 pre-treated Treg (right) (n = 4/group) following overnight activation with plate bound anti-CD3/28 monoclonal antibodies and IL-2. Data show one experiment representative of 2 independent experiments. *p<0.05; **p<0.01; ***p<0.001; ****p<0.0001 by unpaired *t*-test. Error bars=SEM. Median fluorescent intensity=MFI.

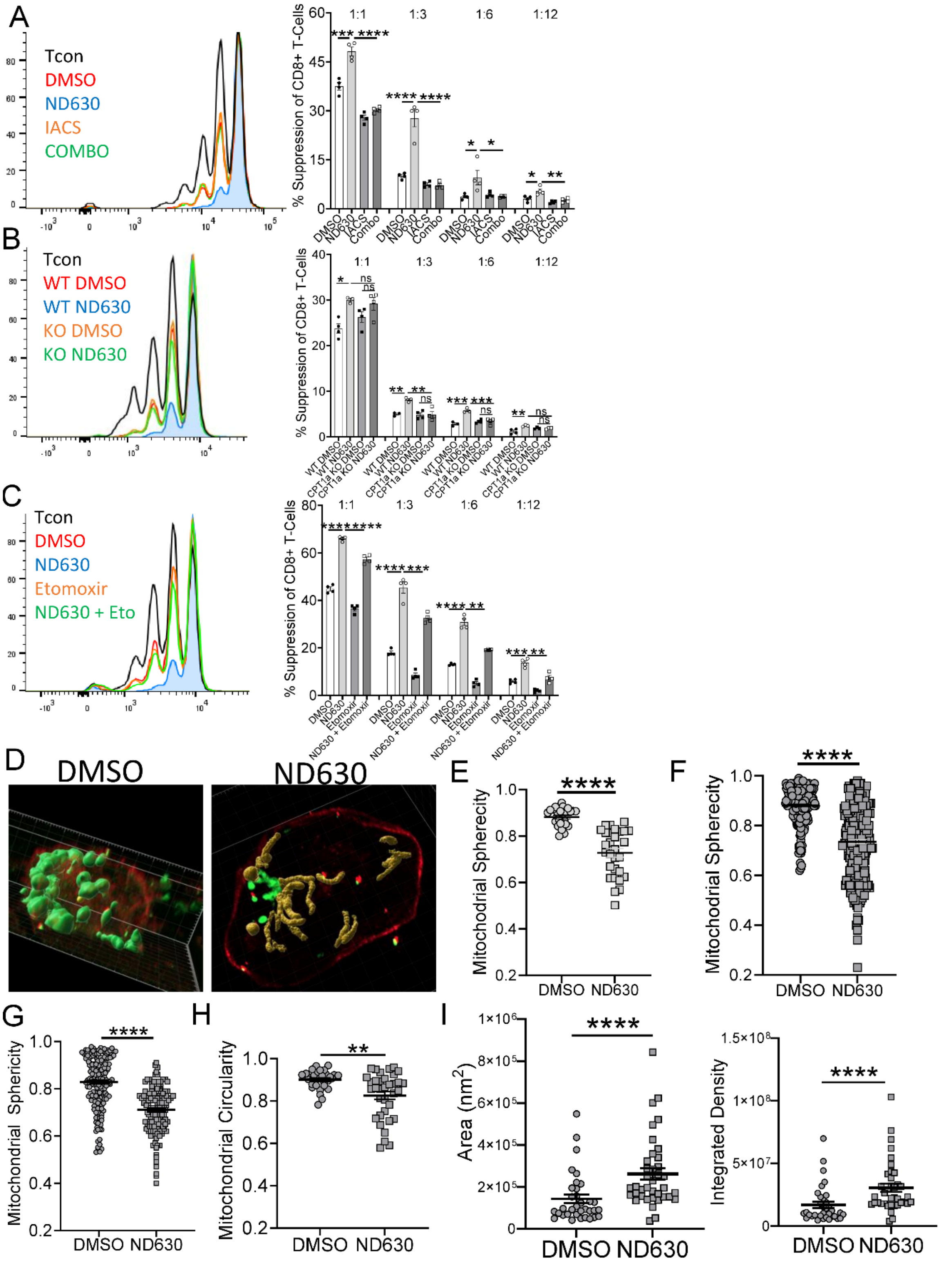

Supplemental Figure 7

Supplemental Figure 7. ACC1 inhibition relies upon OXPHOS and fatty acid utilization for augmentation in Treg function. (A-C) Suppression of CD8+ Tcon proliferation in classical *in vitro* Treg suppression assays by **A)** DMSO, ND630, IACS or ND630+IACS (combo) pre-treated Treg, **B**) WT or CPT1a KO Treg pre-treated with either DMSO or ND630, and **C)** DMSO, ND630, etomoxir or ND630+etomoxir pre-treated Treg. CFSE dilution plots show Treg:Tcon ratio of 1:3. Graphs show 1:1–1:12 Treg:Tcon ratios; n=4/group. (**D-F)** Expansion microscopy analysis of DMSO or ND630 pre-treated Treg acutely activated on planar lipid bilayers, followed by fixation, staining and image capture. **D)** Representative images of DMSO (left) and ND630 (right) pre-treated Treg with mitochondria denoted in green (DMSO) or orange (ND630). **(E-F)** Quantification of mitochondrial sphericity from images obtained by expansion microscopy, grouped by **E**) total mitochondrial sphericity per cell, with n=25 cells in DMSO, n=29 cells in ND630, or **F**) per mitochondrial (mitochondrial sphericity per mitochondria) with n = 235 mitochondria evaluated in DMSO group and n = 286 evaluated in ND630 group. **G)** Quantification of mitochondrial sphericity per mitochondria of 3D SIM imaging of activated Treg isolated from Dendra2-transgenic mice pre-treated with DMSO or ND630; n=186 mitochondria in DMSO and n=141 in ND630. (**H-I**) Quantification of **H)** mitochondrial circularity, **I)** mitochondrial area and mitochondrial integrated density per mitochondria from electron microscopy imaging of overnight activated DMSO and ND630 pre-treated Treg; n=35 mitochondria in DMSO; n=38 mitochondria in ND630. Data show one experiment representative of 2 (expansion microscopy, electron microscopy) or 3 independent experiments. *p<0.05; **p<0.01; ***p<0.001; ****p<0.0001 by unpaired *t*-test, or one way ANOVA. Error bars=SEM.

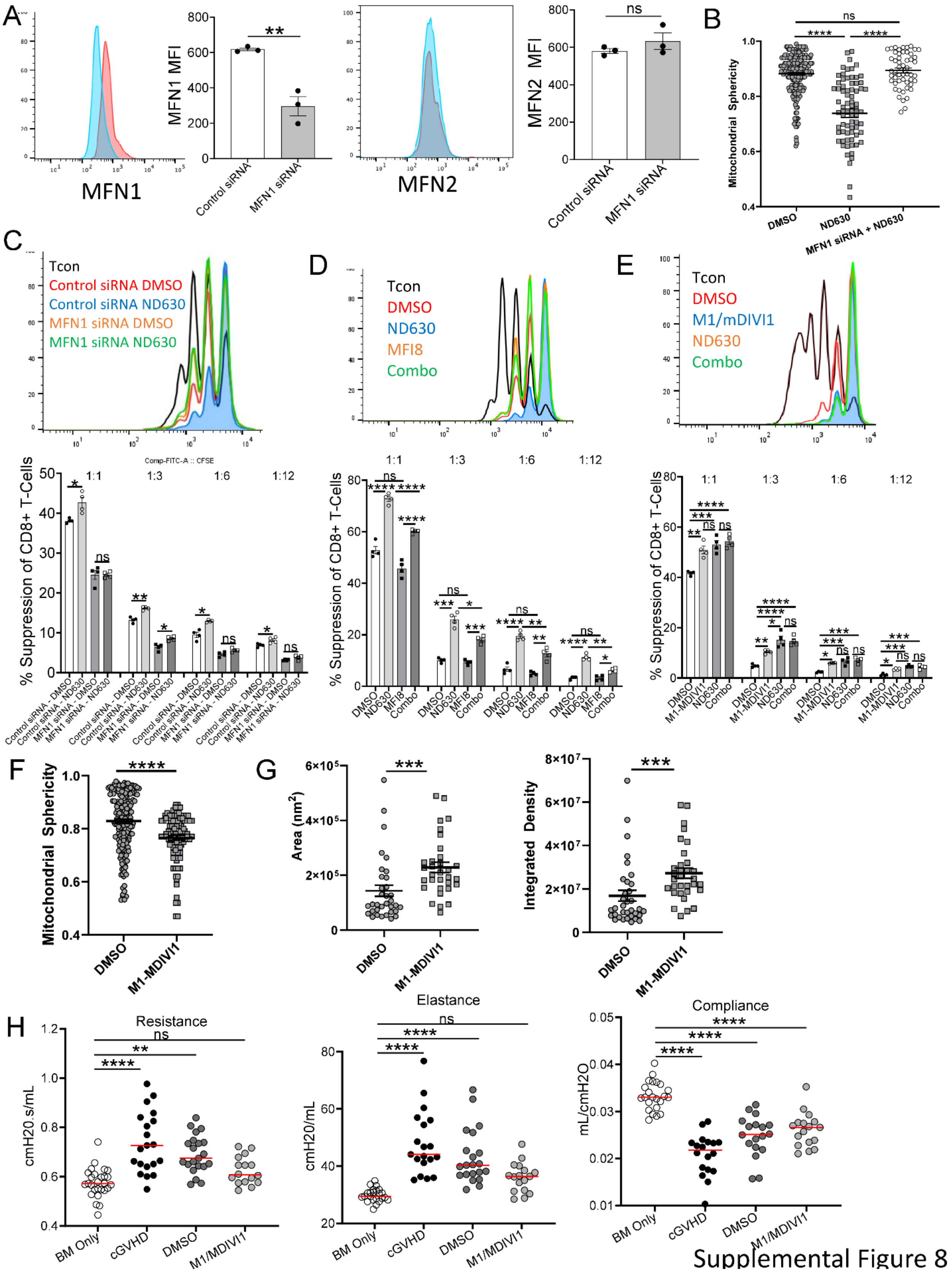

Supplemental Figure 8

Supplemental figure 8. Mitochondrial fusion is critical for Treg suppressive function. A) Flow cytometry analysis of the expression of MFN1 (left) and MFN2 (right) after control or MRN1 siRNA transfection; n = 3/group. **B)** Quantification of mitochondrial sphericity of 3D SIM imaging of control siRNA or MFN1 siRNA transduced Dendra2 Treg pre-treated with ND630; n=235 control siRNA+DMSO (DMSO), n=75 control siRNA+ND630 (ND630) group, and n=52 MFN1 siRNA+ND630 group. **(C-E)** Suppression of CD8+ Tcon proliferation in classical *in vitro* Treg suppression assays by **C)** control siRNA and MFN1 siRNA transduced Treg pre-treated with either DMSO or ND630, **D**) DMSO, ND630, MFI8 and ND630+MFI8 (combo) pre-treated Treg, and **E)** DMSO, M1/MDIVI1, ND630, or ND630+M1/MDIVI1 (combo) pre-treated Treg. CFSE dilution plots show Treg:Tcon ratio of 1:3. Graphs show 1:1–1:12 Treg:Tcon ratios; n=4/group. **F)** Quantification of mitochondrial sphericity from 3D SIM imaging of activated Treg isolated from Dendra2 transgenic mice pre-treated with DMSO or M1/MDIVI1; n=183 DMSO; n=103 M1/MDIVI1. **G)** Quantification of mitochondrial area and mitochondrial integrated density from electron microscopy imaging analysis of overnight activated DMSO and M1/MDIVI1 pre-treated Treg; n=34 DMSO; n=32 ND630. **H)** Chronic GVHD with B10.BR recipients receiving BM± B6 T-cells (D0). Two groups of mice given BM+T-cells, were subsequently given DMSO or M1/MDIVI1 pre-treated Treg on D28 post-transplant. Pulmonary function tests obtained on D49 post-transplant showing Airway Resistance, Lung Elastance and Total Lung Compliance of mice given either BM alone, BM+T-cells (cGVHD), or BM+T-cells and either DMSO or M1/MDIVI1 pre-treated Treg on D28. Data are pooled from 3 transplants; n=21 BM only; n=18 cGVHD; n=19 DMSO Treg; n=17 M1/MDIVI1 Treg. Data show one experiment representative of 3 or 4 (MFN1/2 flow

cytometry) independent experiments. *$p<0.05$; **$p<0.01$; ***$p<0.001$; ****$p<0.0001$ by unpaired *t*-test, or one-way ANOVA. Error bars=SEM.

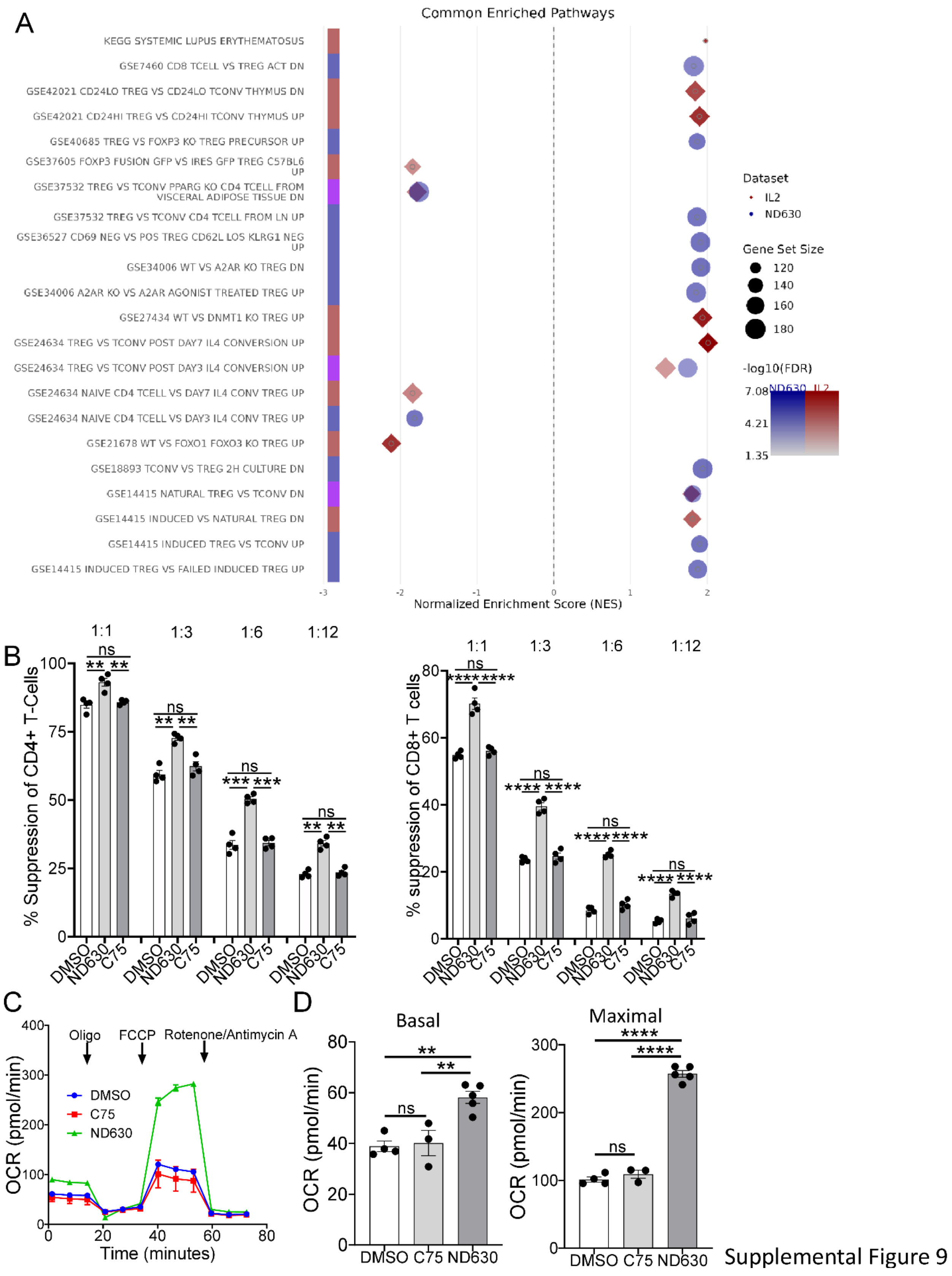

Supplemental Figure 9

Supplemental Figure 9. ACC1 inhibition gene signature is found in human Treg. A) Bulk RNA sequencing results showing overlapping gene signatures between *in vitro* mouse Treg treated with DMSO vs ND630, and human Treg purified from patients with chronic GVHD treated with IL-2 therapy (IL-2 responders vs non-responders). **A)** GSEA bubble plot showing pathway enrichment comparisons between the data set analyzed in mouse Treg with DMSO vs ND630, and bulk RNA sequencing from Treg isolated from patients with chronic GVHD treated low-dose IL-2. Three individual samples were collected and analyzed in the DMSO group, while 4 were collected and analyzed in the ND630 group. For human data, 6 patient samples were collected and analyzed from the IL-2 responder group, and 5 from the non-responder group. Differentially expressed genes defined as having an adjusted value of $P < 0.05$, and a $\log_2$ fold change greater than 0.15. (**B)** Suppression of CD4+ and CD8+ Tcon proliferation in classical *in vitro* Treg suppression assays by DMSO, C75 vs. ND630 pre-treated Treg. 1:1–1:12 Treg:Tcon ratios denoted. (**C-D)** OCR analysis in Seahorse mitochondrial stress test assays, comparing overnight activated DMSO, C75 and ND630 pre-treated Treg, with **C)** OCR curve and **D)** quantification of basal and maximal OCR; n=4 for DMSO, n=3 for C75 and and n=5 for ND630. Data show one experiment representative of 2 (C75 analysis) independent experiments. *p<0.05; **p<0.01; ***p<0.001; ****p<0.0001 by unpaired *t*-test or one-way ANOVA. Error bars=SEM.
